# SARS-CoV-2 spike-ferritin-nanoparticle adjuvanted with ALFQ induces long-lived plasma cells and cross-neutralizing antibodies

**DOI:** 10.1038/s41541-023-00638-6

**Published:** 2023-03-18

**Authors:** Shikha Shrivastava, Joshua M. Carmen, Zhongyan Lu, Shraddha Basu, Rajeshwer S. Sankhala, Wei-Hung Chen, Phuong Nguyen, William C. Chang, Jocelyn King, Courtney Corbitt, Sandra Mayer, Jessica S. Bolton, Alexander Anderson, Isabella Swafford, Guillermo D. Terriquez, Hung V. Trinh, Jiae Kim, Ousman Jobe, Dominic Paquin-Proulx, Gary, R. Matyas, Gregory D. Gromowski, Jeffrey R. Currier, Elke Bergmann-Leitner, Kayvon Modjarrad, Nelson L. Michael, M. Gordon Joyce, Allison M. W. Malloy, Mangala Rao

**Affiliations:** 1 Laboratory of Adjuvant and Antigen Research, U.S. Military HIV Research Program, Walter Reed Army Institute of Research, Silver Spring, MD USA; 2 Henry M. Jackson Foundation for the Advancement of Military Medicine, Bethesda, MD USA; 3 Center for Infectious Disease Research, Walter Reed Army Institute of Research, Silver Spring, MD USA; 4 Department of Pediatrics, F. Edward Hebert School of Medicine, Uniformed Services University of the Health Sciences, Bethesda, MD USA; 5 Emerging Infectious Diseases Branch, Walter Reed Army Institute of Research, Silver Spring, MD USA; 6 Viral Diseases Branch, Walter Reed Army Institute of Research, Silver Spring, MD USA; 7 Malaria Biologics Branch, Walter Reed Army Institute of Research, Silver Spring, USA; 8 Oak Ridge Institute of Science and Education, Oak Ridge, TN 37831 USA

**Keywords:** Vaccines, Infectious diseases

## Abstract

This study demonstrates the impact of adjuvant on the development of T follicular helper (Tfh) and B cells, and their influence on antibody responses in mice vaccinated with SARS-CoV-2-spike-ferritin-nanoparticle (SpFN) adjuvanted with either Army Liposome Formulation containing QS-21 (SpFN + ALFQ) or Alhydrogel^®^ (SpFN + AH). SpFN + ALFQ increased the size and frequency of germinal center (GC) B cells in the vaccine-draining lymph nodes and increased the frequency of antigen-specific naive B cells. A single vaccination with SpFN + ALFQ resulted in a higher frequency of IL-21-producing-spike-specific Tfh and GC B cells in the draining lymph nodes and spleen, S-2P protein-specific IgM and IgG antibodies, and elicitation of robust cross-neutralizing antibodies against SARS-CoV-2 variants as early as day 7, which was enhanced by a second vaccination. This was associated with the generation of high titer, high avidity binding antibodies. The third vaccination with SpFN + ALFQ elicited high levels of neutralizing antibodies against the Omicron variant. No cross-neutralizing antibodies against Omicron were induced with SpFN + AH. These findings highlight the importance of ALFQ in orchestrating early induction of antigen-specific Tfh and GC B cell responses and long-lived plasma cells in the bone marrow. The early engagement of S-2P specific naive B cells and high titer IgM antibodies shape the development of long-term neutralization breadth.

## Introduction

The emergence of SARS-CoV-2, the coronavirus responsible for the global COVID-19 pandemic, resulted in an unprecedented and accelerated quest for a safe and efficacious vaccine^1^. As of March 2023, there are 183 vaccines in clinical development and 199 vaccines in pre-clinical development with an ever-changing landscape of candidate vaccines^2^. Several vaccines targeting the whole virus or the spike protein which also includes the receptor binding domain (RBD) were produced and are being used worldwide^3,4^. These include mRNA, adenovirus vectored, whole virus inactivated, and protein subunit vaccines^5,6^. In the U.S., four COVID-19 vaccines have been authorized for emergency use (EUA) or FDA-approved. Two of the approved vaccines are based on mRNA platforms, the third EUA vaccine is based on an adenoviral vector^7–9^, and the fourth EUA vaccine, Novavax COVID-19 Vaccine, contains the SARS-CoV-2 spike protein and Matrix-M adjuvant^10,11^. The approved/authorized vaccines were effective against the original SARS-CoV-2 strain but immunity was short-lived and required booster vaccinations^12^. In addition, the rapid evolution of the original SARS-CoV-2 strain and the global circulation of emerging new variants of concern (VOC) such as Alpha (B.1.1.7), Beta (B.1.351), Gamma (P.1), Delta (B.1.617.2), and Omicron (BA.1; BA.2; BA.2.12.1 and BA.4/5), continues to cause surges in COVID-19 infections and deaths prompting the development of new vaccines using the identified mutations in the spike protein of VOC^13–16^. The lack of durable, cross-protective immune responses elicited with approved vaccines, coupled with the risk of vaccine escape by newly emerging variants is a constant threat^17^, that has created unique challenges for the development of the next generation of COVID-19 vaccines.

We recently developed a SARS-CoV-2 sub-unit vaccine based on a ferritin nanoparticle platform that displays a prefusion stabilized spike on its surface (SpFN)^18^. The SpFN vaccine formulated with a potent adjuvant, Army Liposome Formulation containing QS-21 (ALFQ) was previously evaluated in several pre-clinical studies and induced high titer antibody responses, polyfunctional T cell responses, enhanced recruitment and activation of classical and non-classical antigen-presenting cells (APCs) in the vaccine draining lymph nodes of mice, leading to durable SARS-CoV-2 specific adaptive T cell immune responses, and conferred protective immunity against challenge with VOC in hamsters and non-human primates^19–22^. In addition, the vaccine-induced protection of K-18 hACE2 transgenic mice passively transferred with IgG antibodies generated by SpFN + ALFQ vaccine^19–22^. However, the mechanism of action of ALFQ in inducing potent immune responses remains unclear.

ALFQ consists of saturated phospholipids, cholesterol, and two immunostimulants, synthetic monophosphoryl lipid A and the saponin QS-21^23,24^. Based on the highly efficacious and potent immune responses generated by this vaccine in preclinical studies, the vaccine was evaluated for the safety and immunogenicity in a phase 1 clinical trial (ClinicalTrials.gov Identifier: NCT04784767). The trial has been completed and the vaccine was found to be safe. The immunogenicity data are currently being analyzed.

In this study, we evaluated the induction of T follicular helper (Tfh) cell and B cell-mediated humoral immune responses in C57BL/6 mice vaccinated with SpFN adjuvanted with ALFQ (SpFN + ALFQ) and compared the responses with SpFN formulated with Alhydrogel^®^ (SpFN + AH). A single vaccination with SpFN + ALFQ rapidly induced spike-specific IL-21-producing Tfh cells and germinal center (GC) B cells associated with significantly higher magnitude antibody responses as early as day 5, which was enhanced upon additional vaccinations. SpFN + ALFQ vaccination also elicited long-lived plasma cells in the bone marrow. Analysis of the elicited antibodies demonstrated greater Fc effector function and broad neutralization of the parental SARS-CoV-2 strain and four VOC (Alpha, Beta, Delta, and Omicron).

## Results

### Mice vaccination

The stabilized spike trimer-ferritin fusion recombinant protein nanoparticle (SpFN) was designed using the initial SARS-CoV-2 genome sequence from Wuhan, China (GenBank:MN9089473). The design, characterization, and immunogenicity of SpFN formulated with Alhydrogel^®^ (AH, SpFN + AH) and with the saponin-liposome formulation, ALFQ (SpFN + ALFQ) have been previously described^18–22^. C57BL/6 mice were vaccinated intramuscularly at three-week intervals on days 0, 21, 42 or weeks 0, 3, and 6, with either SpFN + AH or SpFN + ALFQ. The various immunogenicity assays were carried out on days 3, 5, 7, 10, 14, 21, 28, 42, 56, 70, 112, and 147.

### SpFN + ALFQ vaccine-induced robust T follicular helper (Tfh) cells and polyfunctional spike-specific CD4 T cells in the draining lymph nodes (dLN)

**Fig. 1:**
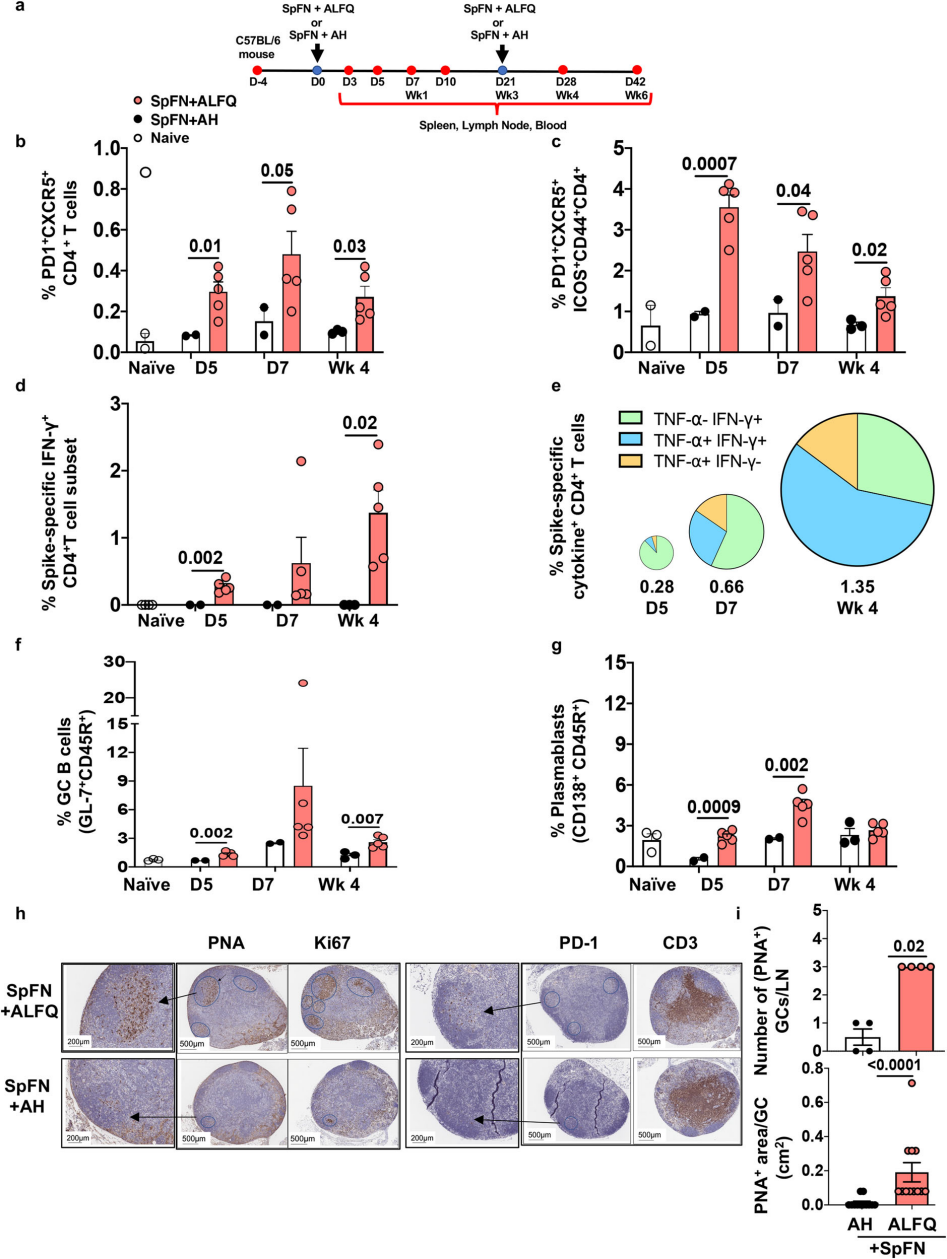
SpFN + ALFQ induces higher frequencies of T follicular helper (Tfh) cells, polyfunctional spike-specific CD4^+^ T cells, and germinal center (GC) B cells in the draining lymph nodes (dLN). **a** Timeline of vaccination and sample collection. C57BL/6 mice were vaccinated with SARS-CoV-2 antigen (SpFN) formulated with a saponin containing liposomal adjuvant formulation, ALFQ (SpFN+ALFQ) or adsorbed to aluminum hydroxide, AH (SpFN+AH) on days 0 and 21 (weeks 0 and 3) as indicated (blue dot). Blood and tissues (red dots) were collected at varying timepoints for immunological analysis. dLNs were collected from naive and vaccinated mice on days 5, 7, and 28 (week 4). Single cell suspensions were made and were stimulated with peptide pools covering the full length of the spike protein. The frequencies of spike-specific Tfh and GC B cells were determined at each time point by flow cytometry. Open and closed (black and salmon color) circles represent dLN cells from naive, SpFN + AH, and SpFN + ALFQ mice, respectively. **b** Frequencies of Tfh (PD-1^+^CXCR5^+^CD4^+^ T cells), **c** (PD-1^+^CXCR5^+^ICOS^+^CD44^+^ CD4^+^ T cells). **d** Percentage of IFN-γ expressing spike-specific CD4^+^ T cells. **e** Pie chart showing the percentage of polyfunctional spike-specific CD4^+^ T cells from SpFN + ALFQ vaccinated mice expressing IFN-γ and/or TNF-α. The slices of the pie and each slice represent the frequencies of cytokine expression in CD4^+^ T cells. The numbers below each pie chart represent the percentage of spike-specific CD4^+^ T cells expressing IFN-γ and/or TNF-α at the indicated time points. **f** Percentage of germinal center B cells (GL7^+^ CD45R^+^). **g** Percentage of plasmablasts (CD45R^+^CD138^+^) (*n* = 5 mice/group/time point in vaccination groups; *n* = 3 mice in control group); SpFN + AH dLNs were pooled into two samples for days 5 and 7 and into three samples for week 4. The flow panels and the gating strategy for the respective subsets are shown in Supplementary Tables 1 and 2, and Supplementary Fig. 1, respectively. For Immunohistochemistry (IHC) analysis (**h**) vaccine dLNs were obtained on week 4 from each of the vaccination groups. Sections were stained with anti-PNA, anti-Ki67, anti-PD-1, and anti-CD3. Follicular structures that were CD3^+^PD-1^+^ T follicular helper (Tfh) and PNA^+^ and Ki67^+^ were considered as germinal centers. Images were analyzed with Histowiz platform. The areas positive for the PNA, Ki67, and PD-1 were marked for ease of counting and measurement of the area. Arrows depict the GCs in the lymph node and an enlarged area of that region is shown. **i** Each lymph node was analyzed for the number of GCs (PNA^+^) and the area of each GC in a given lymph node and divided by the number of lymph nodes or GCs in each group. The data are represented as bar graphs (Mean ± SEM) and each dot represents the average number of GCs per dLN or the area of individual GC per dLN. The statistical differences between the two vaccinated groups were evaluated by Mann–Whitney *U*-test with *P* ≤ 0.05 considered as significant. The scale bars represent 200 and 500 μM.

Tfh cells are essential for the formation and maintenance of GCs, providing specialized help to GC B cells that undergo selection and differentiation into plasmablasts, memory B cells, and class-switched high-affinity antibody-secreting long-lived plasma cells (LLPCs)^25,26^. Tfh (PD1^hi^CXCR5^+^ICOS^+^CD44^+^CD4^+^) cells from the vaccine draining inguinal and popliteal lymph nodes from SpFN + ALFQ or SpFN + AH vaccinated mice were analyzed. The immunization strategy and biospecimen collection are shown in Fig. 1a. The flow cytometry antibody panel and gating strategy are shown in Supplementary Table 1 and Supplementary Fig. 1a. A significant increase of Tfh in the dLN was seen on days 5, 7, and week 4 post SpFN + ALFQ vaccination. This was in contrast to SpFN + AH vaccinated mice where Tfh frequencies were similar to unvaccinated mice (Fig. 1b, c).

We further determined the frequency of cytokine-expressing spike-specific T cells in the dLNs of vaccinated mice by intracellular cytokine staining (ICS) following stimulation with two peptide pools covering the full-length of the spike protein. The frequency of cytokine-expressing spike-specific T cells in SpFN + AH vaccinated mice were too low to be reliably identified by flow cytometry. In contrast, functional spike-specific T cells in SpFN + ALFQ vaccinated mice were identified by the expression of IFN-γ and/or TNF-α on days 5 (0.28%) and 7 (0.66%) and were further increased in frequency at week 4 (1.35%) (Fig. 1d, e; Supplementary Fig. 1b). The spike-specific CD4^+^T cells induced by SpFN + ALFQ vaccination were primarily IFN-γ^+^TNF-α^-^ on day 5 but converted to predominantly polyfunctional IFN-γ^+^TNF-α^+^ by week 4 (Fig. 1e). The flow cytometry antibody panel is shown in Supplementary Table 2.

### SpFN + ALFQ vaccine-induced robust plasmablasts and germinal center B cells in the dLN

The germinal center (GC) response is critical for the generation of affinity-matured plasma cells and memory B cells capable of mediating long-term protective immunity. To determine if the induction of Tfh cells by SpFN + ALFQ altered the B cell response, we quantified changes in the frequency of GC B cells and plasmablasts in the dLN. At each experimental timepoint, SpFN + ALFQ vaccination induced more GC B cells (GL-7^+^CD45R^+^) and plasmablasts (CD138^+^CD45R^+^) compared to SpFN + AH vaccination. The frequency of plasmablasts and GC B cells in SpFN + ALFQ vaccination group were significantly higher than the SpFN + AH group and reached maximal frequency at day 7 post first vaccination (Fig. 1f). To further evaluate the evolution of GCs in the dLN, we performed immunohistochemistry (IHC) analysis to visualize the GCs in the dLN at week 4. Tfh cells were identified by staining with antibodies against CD3 and PD-1 and GC B cells were identified by staining with peanut agglutinin (PNA) and Ki67. A global view of dLN sections revealed a greater induction of PNA^+^ or Ki67^+^ GCs B cells and PD-1^+^ Tfh cells by the SpFN + ALFQ vaccine, whereas dLN from the SpFN + AH vaccinated mice showed minimal staining for the GC B cells and Tfh cells (Fig. 1h). The mean number and area of PNA^+^GCs in each of the four dLNs for each formulation was determined and are shown in Fig. 1i. We observed a threefold higher number of GCs with an average area greater than twofold in SpFN + ALFQ vaccinated mice compared to SpFN + AH mice (average area/GC: 0.2cm^2^ vs. 0.08 cm^2^; Fig. 1i). Collectively, these data indicate that SpFN + ALFQ is more effective at inducing Tfh cells and GC B cells as early as day 7 post first vaccination, which was maintained at week 4 (1 week post second vaccination). The increased magnitude of Tfh and GC B cells have been associated with more robust and durable antibody responses^27^.

### SpFN + ALFQ-induced higher frequencies of SARS-CoV-2 specific Tfh and GC B cells in the spleen at day 7

**Fig. 2:**
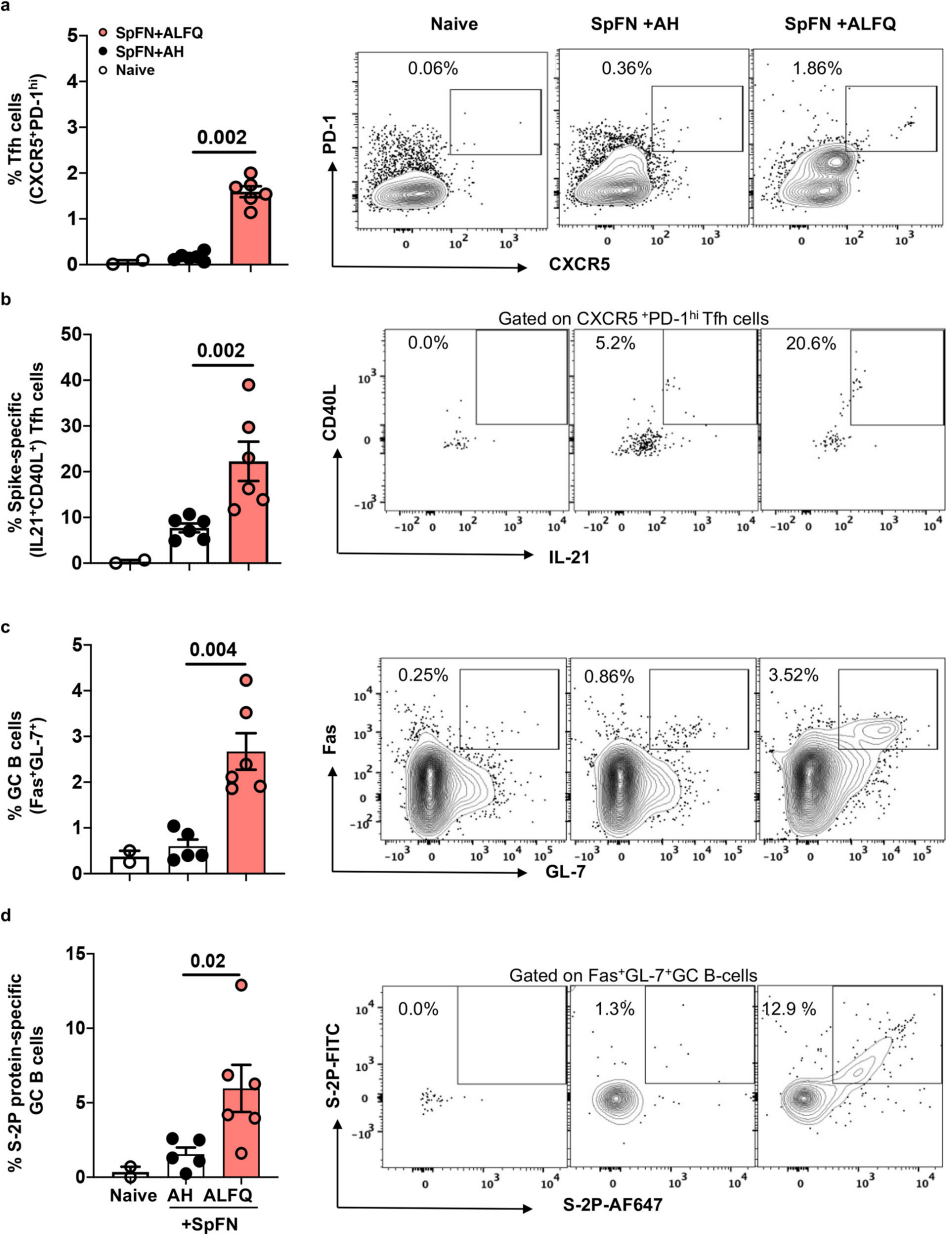
SpFN + ALFQ induces robust spike-specific IL-21-secreting Tfh and S-2P-protein-specific germinal center (GC) B cells in the spleen at day 7. C57BL/6 mice were vaccinated with SpFN + ALFQ or SpFN + AH on day 0 and spleens from vaccinated (*n* = 6 in each group) and naive (*n* = 2) mice were collected on day 7. Splenocytes were stimulated with peptide pools covering the full length of the spike protein and the frequency of **a** Tfh (CXCR5^+^PD-1^hi^) cells and **b** (IL-21^+^ CD40L^+^) spike-specific Tfh cells in all three study groups was determined by ICS (left: naive, center: SpFN + AH, right: SpFN + ALFQ). Flow cytometry analysis was performed to determine the percentage of **c** germinal center (GC; Fas^+^GL-7^+^) B cells and percentage of **d** S-2P protein-specific GC B cells positive for the dual-fluorophore labeled S-2P protein (S-2P-AF647 and S-2P-FITC). The data are represented as bar graphs (Mean±SEM) and each dot represents the data from an individual spleen. The right panels show the representative contour flow plots for each subset. The flow panel and the gating strategy for the respective subsets are shown in Supplementary Tables 3 and 4 and Supplementary Figs. 2 and 3, respectively. The statistical differences between the two vaccinated groups were evaluated by Mann–Whitney *U*-test with *P* ≤ 0.05 considered as significant.

We analyzed the frequencies of Tfh and GC B cells in the spleen at day 7 post first vaccination to determine if the responses recapitulated what we found in the dLN. The flow cytometry antibody panel and gating strategy are shown in Supplementary Table 3 and Supplementary Fig. 2. We noted that a single dose vaccination with SpFN + ALFQ elicited significantly higher frequencies of Tfh cells (CD4^+^CXCR5^+^PD-1^hi^) in the spleen as early as day 7, whereas the SpFN + AH vaccine formulation failed to induce a significant Tfh cell response (Fig. 2a). We further sought to evaluate whether these Tfh cells were antigen-specific by ICS following in vitro stimulation of splenocytes with SARS-CoV-2 spike peptide pools. Functional analysis of Tfh cells demonstrated that SpFN + ALFQ generated increased spike-specific Tfh cells producing IL-21 and higher frequencies of Fas^+^GL7^+^ GC B cells compared to SpFN+AH at day 7 post first vaccination (Fig. 2b, c). To determine if these GC B cells were antigen-specific, SARS-CoV-2 spike (S-2P) protein was separately conjugated with either FITC or Alexa Fluor^®^647 and used as probes for flow cytometry-based detection of antigen-specific GC B cells. We observed greater than a fourfold higher frequency of S-2P protein-specific GC B cells in SpFN + ALFQ vaccinated mice compared to SpFN + AH (Fig. 2d). Representative flow plots depicting the differences in the frequency of the respective cells are shown in the right panels. The flow panel and the gating strategy for Tfh, IL-21-secreting Tfh, GC B cells, and spike-specific GC B cells are shown in Supplementary Tables 3 and 4; Supplementary Figs. 2 and 3, respectively.

### SpFN + ALFQ enhanced SARS-CoV-2-spike-specific Tfh and GC B cell responses in the spleen at week 4

**Fig. 3:**
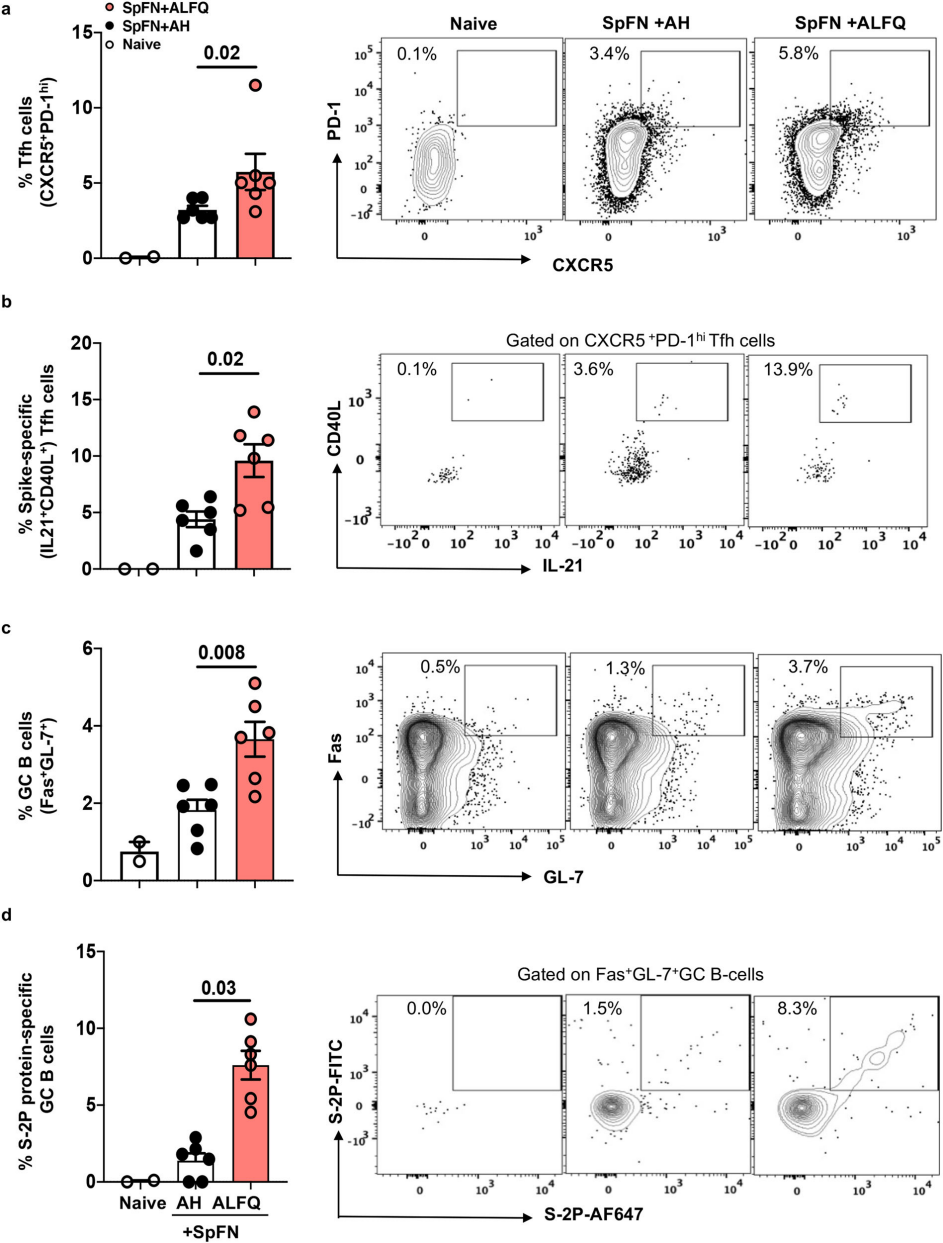
SpFN + ALFQ vaccine enhanced the frequency of T follicular helper (Tfh) cells, spike-specific IL-21-secreting Tfh cells and S-2P protein-specific germinal center (GC) B cells in the spleen following second vaccination (week 4). C57BL/6 mice were vaccinated with SpFN+ALFQ or SpFN+AH on days 0 and 21 (week 3). Spleens from vaccinated (*n* = 6 in each group) and naive (*n* = 2) mice were collected on week 4 and flow cytometry analysis was performed. The frequency of **a** Tfh (CXCR5^+^PD-1^hi^) cells in all three groups, **b** IL-21^+^CD40L^+^ spike-specific Tfh cells, **c** GC (Fas^+^GL-7^+^) B cells and **d** S-2P protein-specific GC B cells positive for the dual-fluorophore (S-2P-AF647 and S-2P-FITC) are shown. The data are represented as bar graphs (Mean ± SEM) and each dot represents the data from an individual spleen. The right panels show the representative contour flow plots for each subset. The flow panel and the gating strategy for the respective subsets are shown in Supplementary Tables 3 and 4 and Supplementary Figs. 2 and 3, respectively. The statistical differences between the two vaccinated groups were evaluated by Mann–Whitney *U*-test with *P* ≤ 0.05 considered as significant.

To investigate the evolution of Tfh and GC B cell responses, we analyzed spleen cells at week 4 (one week post second vaccination). Our data demonstrated that two vaccinations with SpFN + ALFQ induced significantly greater frequencies of Tfh and spike-specific IL-21-producing Tfh cells (Fig. 3a, b) as well as a significant increase in Fas^+^GL7^+^ GC B cells (Fig. 3c). Furthermore, we observed that two vaccinations with SpFN + ALFQ generated significantly higher S-2P protein-specific GC B cells (Fig. 3d) compared to SpFN + AH. Representative flow plots depicting the differences in the frequency of the respective cells are shown in the right panels. The flow panel and the gating strategy for the Tfh, IL-21-secreting Tfh cells, GC B cells, and spike-specific GC B cells are shown in Supplementary Tables 3 and 4; Supplementary Figs. 2 and 3, respectively.

### SpFN + ALFQ induced higher frequencies of S-2P protein-specific naive B cells and IgG1^+^ memory B cells

**Fig. 4:**
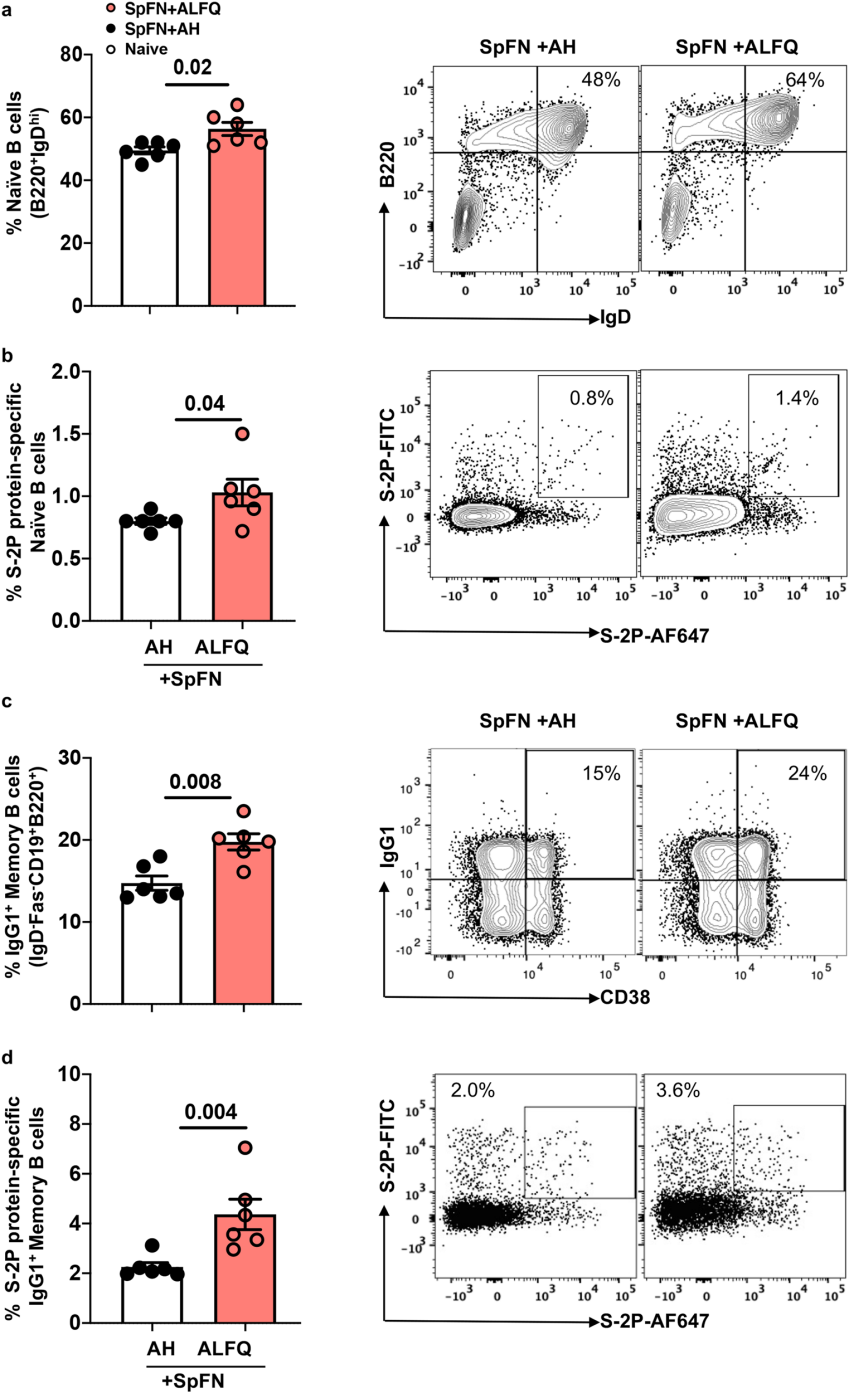
SpFN + ALFQ vaccinated mice elicited higher frequencies of S-2P protein-specific naive B cells and S-2P protein-specific-IgG1^+^ memory B cells (MBCs) in the spleen following priming vaccinations. Spleens from vaccinated (*n* = 6 in each group) and naive (*n* = 2) mice were collected on week 4. Flow cytometry analysis was performed to determine the frequencies of naive B cells and MBCs. **a** Frequency of naive B (CD19^+^B220^+^IgD^hi^) cells in all the three groups. **b** Frequency of S-2P protein-specific naive B (CD19^+^B220^+^IgD^hi^) cells. **c** IgG-1^+^CD38^+^MBCs (IgD^-^Fas^-^CD19^+^B220^+^) and **d** S-2P protein-specific IgG-1^+^MBCs are shown. The data are represented as bar graphs (Mean±SEM) and each dot represents the data from an individual spleen. The right panels show the representative contour flow plots for each subset. The flow panel and the gating strategy for the respective subsets are shown in Supplementary Table 4 and Supplementary Figs. 4 and 5a, respectively. The statistical differences between the two vaccinated groups were evaluated by Mann–Whitney *U*-test with *P* ≤ 0.05 considered as significant.

It has been recently reported that vaccine-induced potent cross-neutralizing humoral immune responses are dependent on the recruitment, activation, and maturation of antigen-specific naive B cells^28^. Therefore, we assessed the differences in the frequencies of naive B cells (CD19^+^B220^+^IgD^hi^) and S-2P protein-specific naive B cells in response to SpFN + ALFQ and SpFN + AH vaccines. The frequencies of naive B cells at week 4 and S-2P protein-specific naive B cells at week 4 were significantly higher in the spleen of SpFN + ALFQ vaccinated mice compared to SpFN + AH (Fig. 4a, b). Memory B cells (MBCs) persist and rapidly differentiate into antibody-secreting cells upon the antigen re-encounter. MBCs re-enter the GCs and undergo further affinity maturation and have the ability to recognize and respond to viral variants^29^. We therefore assessed the frequency of IgG1^+^CD38^+^MBCs in the vaccinated mice. The gating strategy for the naive and S-2P protein-specific naive B cells, MBCs and S-2P-protein-specific IgG1^+^ MBCs are shown in Supplementary Fig. 4 and Supplementary Fig. 5a, respectively. Representative flow plots depicting the differences in the frequency of the respective cells are shown in the right panels. The flow panel is shown in Supplementary Table 4. We noted a significantly higher frequency (greater than twofold) of IgG1^+^CD38^+^MBCs in SpFN + ALFQ vaccinated mice compared to SpFN + AH (Fig. 4c, d). This increase was also reflected in a four-fold higher amount of S-2P protein-specific IgG1 antibodies in the sera of SpFN + ALFQ vaccinated mice (73.10 µg/mL) compared to SpFN + AH vaccinated mice (18.04 µg/mL) (Supplementary Fig. 5b).

### SpFN + ALFQ-induced greater frequencies of plasmablasts, spike-specific IgG and IgA secreting cells and long-lived plasma cells

**Fig. 5:**
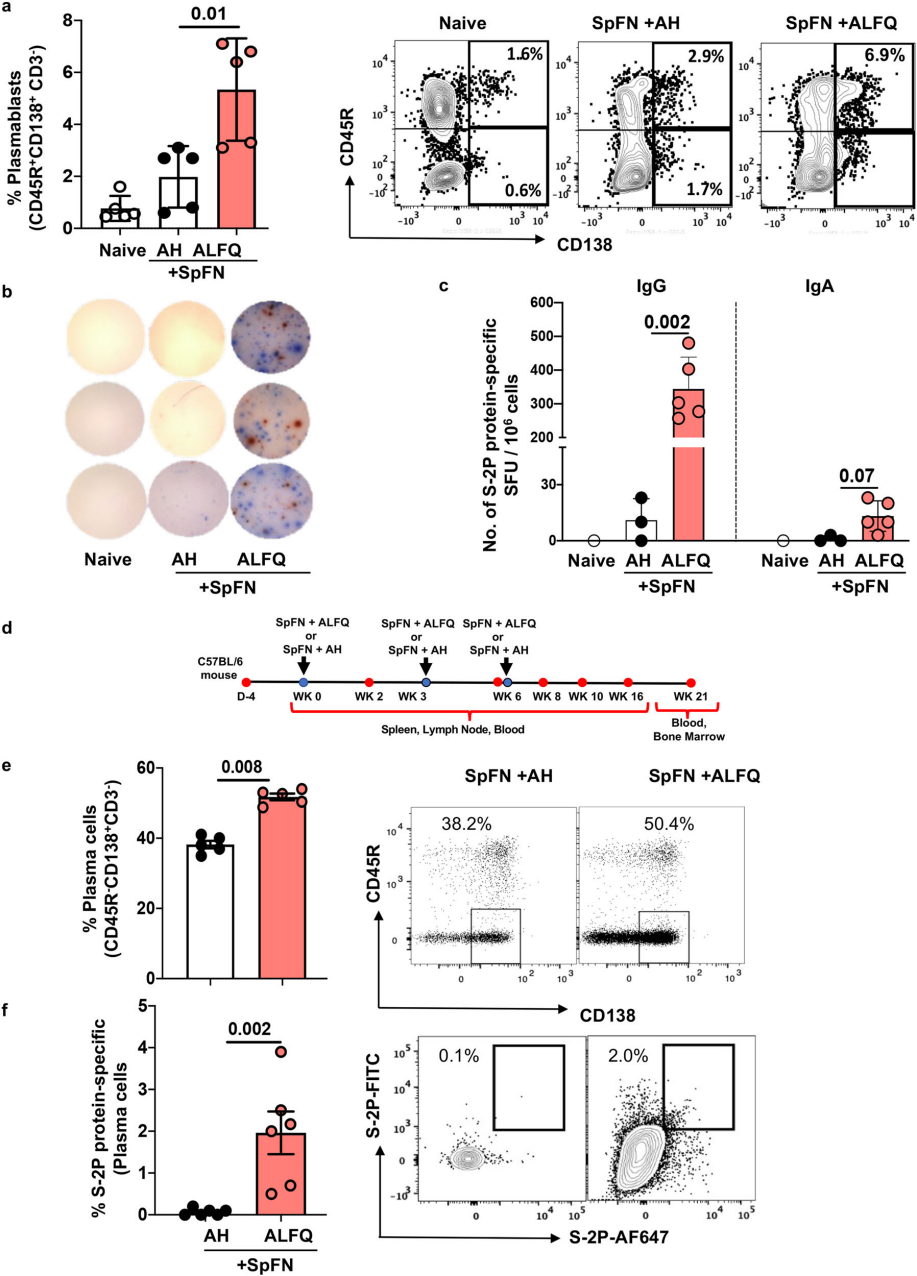
SpFN + ALFQ-induced significantly higher frequencies of plasmablasts in the spleen and higher frequencies of S-2P protein-specific long-lived plasma cells (LLPCs) in the bone marrow. Spleens from naive, or vaccinated SpFN + ALFQ or SpFN + AH mice (*n* = 5 in each group) were collected at week 6 (day 42, Fig. 5d). ELISpot and flow cytometry analysis were performed. **a** Percentage of plasmablasts (CD3^-^CD45R^+^CD138^+^) in all the three groups. **b** ELISpot images of the triplicate wells showing S-2P protein-specific- IgG (blue spots) and IgA (red spots) for each group. **c** Number of S-2P protein-specific IgG and IgA spot-forming units per million splenocytes are presented as a bar graph (Mean ± SEM). Each dot in the bar graph represents the average of triplicate wells for each mouse in each group. The responses were considered positive when the spot count exceeded the Mean ± 3 SD of the negative control wells. **d** A separate group of mice (*n* = 6 in each group) were vaccinated three times with SpFN + ALFQ or SpFN + AH. Bone marrows were collected at week 21 and the percentage of **e** plasma cells (CD3^-^CD45R^-^CD138^+^) and **f** intracellular S-2P protein-specific plasma cells were analyzed. The data are represented as bar graphs (Mean ± SD) and each dot represents the data from an individual spleen or bone marrow. The right panels show the representative contour or dot plots for each subset. The flow panel and the gating strategy for the respective subsets are shown in Supplementary Table 5 and Supplementary Fig. 6, respectively. The statistical differences between the two vaccinated groups were evaluated by Mann–Whitney *U*-test with *P* ≤ 0.05 considered as significant.

The frequency of plasmablasts (CD3^-^CD45R^+^CD138^+^) in the spleen of SpFN+ALFQ vaccinated mice at week 6 (3 weeks post second vaccination) was twofold higher than SpFN + AH (Fig. 5a). Representative flow plots depicting the differences in the frequency of the plasmablasts are shown in the right panel. The flow panel and the gating strategy are shown in Supplementary Table 5 and Supplementary Fig. 6. To assess the induction of S-2P protein-specific immunoglobulin-secreting cells, splenocytes were incubated with S-2P protein and ELISpot was performed. Representative images of the ELISpot wells illustrating S-2P protein-specific IgG (blue spots) and IgA (red spots) secreting cells are shown (Fig. 5b). S-2P protein-specific IgG and IgA spot-forming units per million (SFC/10^6^) splenocytes from SpFN + ALFQ vaccinated mice were 344 and 19 respectively, compared to 11 and 3 from SpFN + AH mice (Fig. 5c).

Long-lived plasma cells (LLPCs) are terminally differentiated cells that secrete antibodies against previously encountered antigens. LLPCs persist in the bone marrow and serve as a first line of defense against reinfection through the constitutive secretion of antibodies^30–32^. In order to assess the frequency of LLPCs induced by vaccination, a separate group of mice was vaccinated three times at weeks 0, 3, and 6 weeks with SpFN + ALFQ or SpFN + AH. Blood and bone marrow were collected at week 21 (15 weeks post third vaccination; Fig. 5d) and bone marrow cells were analyzed for the presence of LLPCs (CD3^-^CD45R^-^ CD138^+^) by flow cytometry. The flow panel and the gating strategy are shown in Supplementary Table 5 and Supplementary Fig. 6. The frequency of LLPCs was significantly higher in SpFN + ALFQ vaccinated mice compared to SpFN + AH (50.4% vs. 38.2%; *p* = 0.008) (Fig. 5e). The right panel shows the representative flow plots from both groups. Furthermore, the percentage of S-2P protein-specific LLPCs induced by SpFN + ALFQ vaccine was 2.0 % compared to 0.06 % (*p* = 0.002) in SpFN + AH (Fig. 5f) as determined by intracellular staining with S-2P-FITC and S-2P-Alexa Fluor 647.

### SpFN + ALFQ elicited robust RBD and S-2P protein-specific IgM and IgG and cross-neutralizing antibodies

**Fig. 6:**
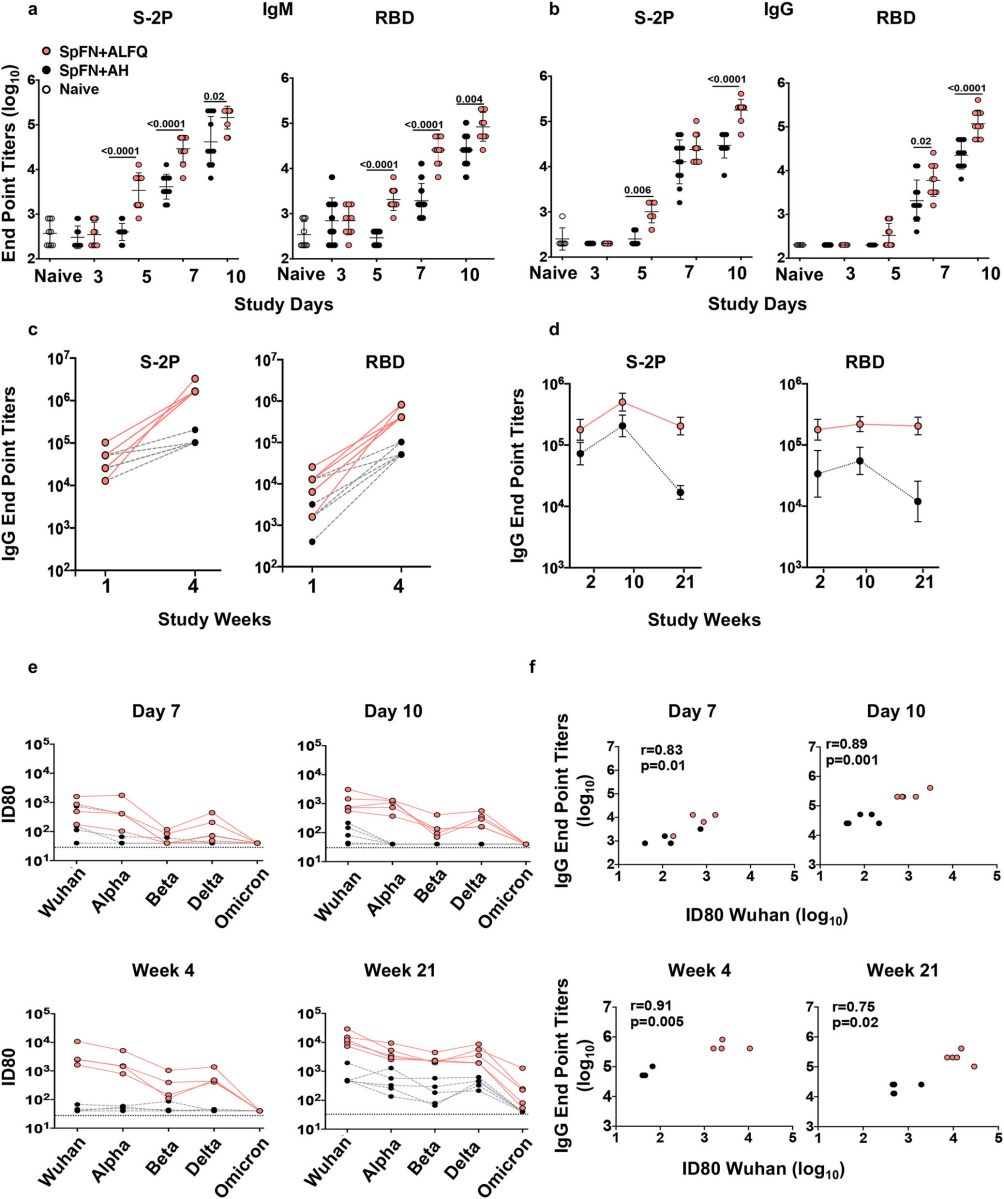
SpFN + ALFQ vaccine elicited rapid and robust antigen-specific binding and neutralizing antibody titers. Sera from C57BL/6 mice vaccinated (Figs. 1a and 5d) with SpFN + ALFQ (*n* = 5 to10) or SpFN + AH (*n* = 5 to 10) or naive mice (*n* = 6) were assessed for binding and neutralizing antibody titers by ELISA and pseudovirus neutralization assays, respectively. **a** IgM and **b**–**d** Serum IgM and IgG antibody responses were assessed against S-2P and RBD proteins on days 3, 5, 7, and 10. In addition, IgG responses were also evaluated on weeks 1, 2, 4, 10, and 21. Data are graphed as Geometric Mean ± Geometric SD. **e** Pseudovirus neutralization titers (ID80) were measured against Wuhan and variants of concern (VOC; Alpha, Beta, Delta, and Omicron) on days 7, 10, and weeks 4 and 21. The horizontal black dotted line represents the lower limit of detection (ID80 = 40) of neutralization titer for the pseudovirus neutralization assay. **f** Spearman correlation between neutralization titers and S-2P protein-specific IgG binding antibody end point titers on days 7, 10, and weeks 4 and 21 are shown. The statistical differences between the two vaccinated groups were evaluated by Mann–Whitney *U*-test with *P* ≤ 0.05 considered as significant.

To evaluate the differences in the antibody kinetics, we measured RBD and S-2P protein-specific binding antibody (IgG and IgM) titers in the sera by enzyme-linked immunosorbent assay (ELISA). In the SpFN + ALFQ vaccinated mice, as early as day 5, there was a significant increase in RBD and S-2P protein-specific IgM and IgG responses compared to mice vaccinated with SpFN + AH (Fig. 6a, b). The geometric mean S-2P protein-specific IgM endpoint titers on day 5 for SpFN + ALFQ was 3408 compared to 400 for SpFN + AH. The IgG endpoint titers for the two groups were 1008 and 252, respectively. The antibody endpoint titers continued to increase exponentially on days 7 and 10 and these titers were further enhanced at week 4 (Fig. 6c). To assess the longitudinal antigen-specific IgG antibody responses, sera from mice vaccinated as shown in Fig. 5d were analyzed. The S-2P and RBD IgG endpoint titers were at least 1 to 1.5 logs higher in SpFN + ALFQ vaccinated mice compared to SpFN + AH vaccinated mice at weeks 2 and 10 (2 and 4 weeks post first and third vaccinations, respectively and the endpoint titers were maintained even at week 21 (15 weeks post 3 vaccination in the SpFN + ALFQ vaccinated mice while they significantly declined in the SpFN + AH vaccinated mice (Fig. 6d).

We further analyzed the sera for the presence of neutralizing antibodies against VOC, Alpha (B.1.1.7), Beta (B.1.351), Delta (B.1.617.2), and Omicron (B.1.1.529). As early as days 7 and 10, the sera of mice vaccinated with SpFN + ALFQ showed significantly higher ID80 neutralization titers against all VOC compared to SpFN + AH except for the Omicron variant (Fig. 6e). The titers were enhanced at weeks 4 and 21 (one week and fifteen weeks post second and third vaccinations, respectively). The ID80 titers for the individual VOC for the early time points (days 7 and 10) and later time points (weeks 4 and 21) are shown in Supplementary Fig. 7a. ID80 neutralization titers against the Omicron variant were detected in the sera of SpFN + ALFQ vaccinated mice at week 21 (Fig. 6e). The ID80 neutralization titers did not show any differences in the two vaccination groups for the Omicron variant at the early time points. The ID50 titers were detected against Omicron at week 4 and were enhanced by at least two logs at week 21 with SpFN + ALFQ vaccine (Supplementary Fig. 7b). In contrast, the ID80 titers for mice vaccinated with SpFN + AH showed low levels of neutralizing antibodies against Wuhan on day 7 which was undetectable by day 28 (week 4). In addition, no neutralizing antibodies (ID80) were detected against the other VOC (Fig. 6e). The ID50 data for sera from SpFN + AH vaccinated mice showed lower levels of neutralizing antibodies against VOC except Omicron variant at the early time points tested. Even after three vaccinations with SpFN + AH, no neutralizing antibodies were detected against the Omicron variant at the time points tested (Supplementary Fig. 7). Furthermore, Spearman correlation analysis showed a high degree of positive correlation between Wuhan pseudovirus ID80 neutralization titers and S-2P-specific IgG end point titers on days 7, 10, and weeks 4 and 21 (Fig. 6f). The correlation plots in Fig. 6f show that higher binding antibody titers were associated with higher neutralization titers. The AH group and the ALFQ groups segregated into two distinct groups with AH exhibiting lower neutralization compared to the ALFQ group.

### SpFN + ALFQ generated high avidity antibodies with greater Fc effector functions

**Fig. 7:**
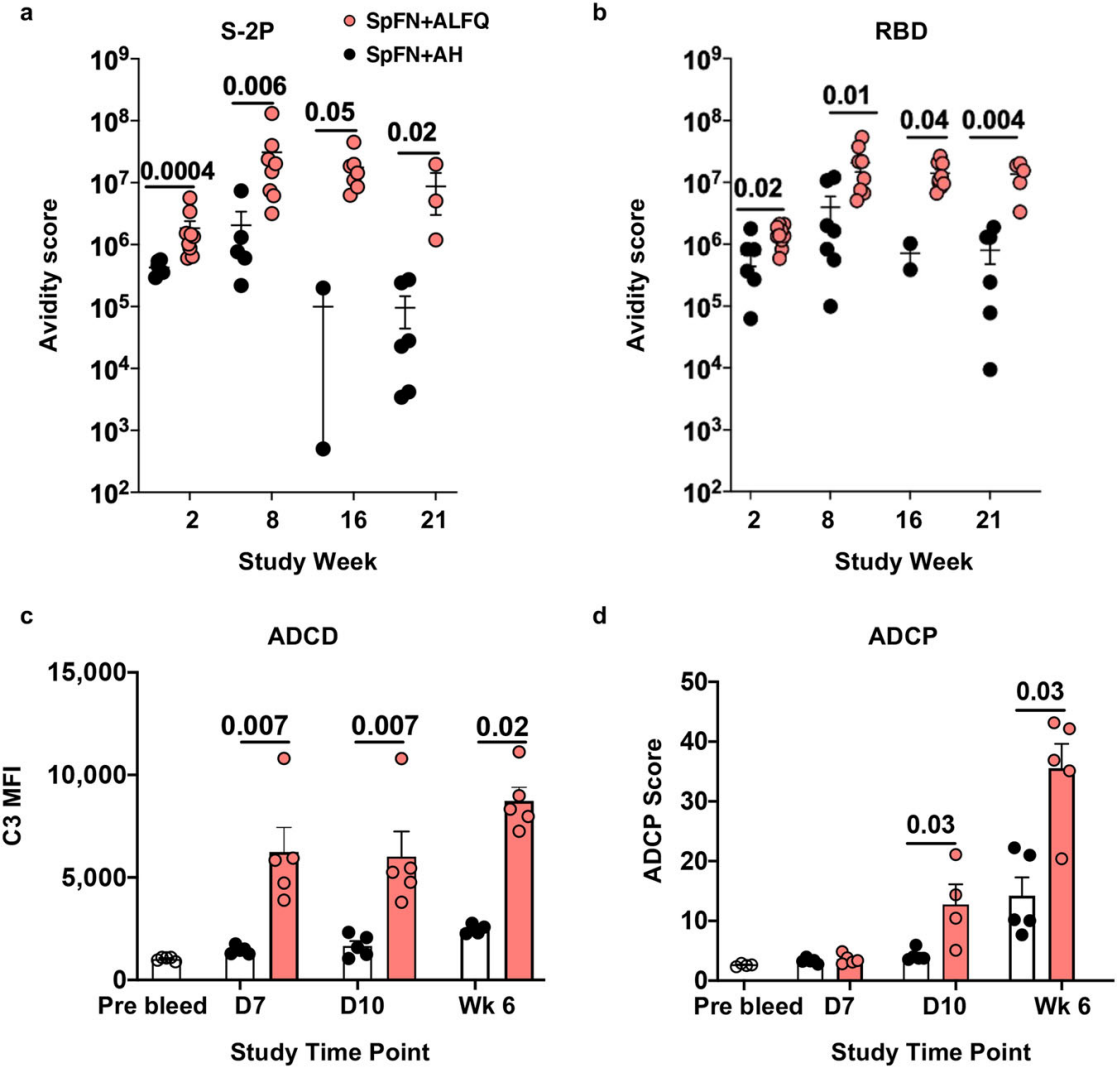
SpFN + ALFQ vaccine-induced antibody avidity maturation and Fc effector functions. Serum from C57BL/6 mice vaccinated (Fig. 5d) with SpFN + ALFQ or SpFN + AH (*n* = 5-10 for each group) was assessed for antibody avidity and antibody Fc effector functions. **a** Surface plasmon resonance data showing S-2P protein-specific and **b** RBD protein-specific antibody avidity score (Response Unit/Kd off rate) at weeks 2, 8, 16, and 21. **c** Antibody-dependent complement deposition (ADCD) and **d** Antibody-dependent cellular phagocytosis (ADCP) activities on days 7, 10 and week 6. Data were graphed as Mean ± SEM. The statistical differences between the two vaccinated groups were evaluated by Mann–Whitney *U*-test using the GraphPad Prism version 9 with **P** ≤ 0.05 considered as significant.

Sera of mice vaccinated with the two formulations were analyzed for antibody avidity by surface plasmon resonance-based binding and kinetic analysis on a BIAcore™ 4000. Avidity maturation as depicted by the Avidity Score (Response units/ Kd off-rate) was observed in both groups against S-2P (Fig. 7a) and RBD (Fig. 7b) proteins starting at week 8. The avidity score against S-2P protein and RBD were significantly decreased at week 16 in the SpFN + AH vaccinated mice (Fig. 7a). In contrast, the avidity score was enhanced at week 16 and maintained at week 21 in the SpFN + ALFQ vaccinated mice (Fig. 7b), thus demonstrating the maintenance of increased antibody avidity.

In addition to mediating neutralization, antibodies can also engage cellular receptors through their Fc region and drive effector functions. We therefore evaluated antibody-dependent complement deposition (ADCD) and antibody-dependent cellular phagocytosis (ADCP) in the sera of mice on days 7, 10, and week 6 following vaccination with SpFN + AH or SpFN + ALFQ. Unlike SpFN + AH vaccinated mice, a significant increase in the ADCD activity was noted as early as day 7, which further increased at week 6 in the SpFN + ALFQ vaccinated mice (Fig. 7c). The sera from both the groups showed ADCP activity. However, the ADCP activity was significantly higher in the ALFQ group compared to the AH group at day 10 and week 6 (Fig. 7d).

## Discussion

We previously reported the development of a self-assembling protein nanoparticle that displays 8 copies of the pre-fusion stabilized SARS-CoV-2 spike glycoprotein trimer, in an ordered array (SpFN) that was adjuvanted with ALFQ^18^. This vaccine formulation showed efficacy against the original SARS-CoV-2 variant (WA1) in K18-hACE2 transgenic mice, Rhesus macaque, and Cynomolgus models and against VOCs Alpha (B.1.1.7) and Beta (B.1.351) in a Syrian golden hamster challenge model^20,22,33^.

Our earlier studies have also demonstrated that the SpFN + ALFQ vaccine-induced early and robust engagement of APCs leading to an increased frequency of polyfunctional SARS-CoV-2 spike-specific CD4 and CD8 T cells with effective cytolytic function and distribution to the lungs^19^. In the current study, we demonstrate that the early induction of Tfh and GC B cell responses by SpFN + ALFQ vaccine contributes to the generation of affinity-matured antibodies with neutralizing activity against VOCs, memory B cells, and long-lived plasma cells.

One of the hallmarks of this study was a single vaccination with SpFN + ALFQ elicited a rapid induction of spike and RBD-specific IgM and IgG antibody responses as early as days 3 and 5, respectively. The IgG responses were further enhanced and consistently maintained after additional vaccinations until the end of the study at week 21. In contrast, the antibody responses with SpFN + AH vaccine were comparatively delayed, and the responses gradually decreased. The early and higher IgM binding titers and the higher frequencies of antigen-specific naive B cells induced by SpFN + ALFQ vaccine may contribute to the development of neutralization breadth. It has been recently reported that induction of high IgM binding titers along with higher frequencies of antigen-specific naive B cells early in acute HIV-1 infection in humans contributes to the development of antibody neutralization breadth^34^.

The binding antibody responses following SpFN + ALQ vaccination exhibited higher avidity to spike and RBD proteins. Longitudinal antibody analysis showed a 10-fold higher avidity, which was maintained even at week 21, while the avidity was not sustained with SpFN + AH vaccination. Previously, it has been demonstrated in a phase 3 RTS,S/AS01E malaria vaccine study, that there is a direct correlation between the antibody concentration, avidity, and the vaccine efficacy^35^. The adjuvant AS01E is also a liposomal formulation containing monophosphoryl lipid A and QS-21 like ALFQ. It is interesting to note that vaccination with SpFN + ALFQ also induced high avidity antibodies. The data from the current study demonstrated that the antibodies induced by SpFN + ALFQ vaccine showed a strong positive correlation between the binding and neutralizing antibodies at the early time points that were maintained out to week 21. The robust neutralizing antibody responses induced were not only against the parental strain but also exhibited cross-neutralization against the VOC; Alpha, Beta, and Delta. Interestingly, high titer cross-neutralization responses were observed as early as day 7 after a single vaccination. Following additional vaccinations, cross-neutralizing antibodies against the Omicron variant were induced. This contrasted with SpFN + AH, which induced 2–3 log lower levels of neutralizing antibody responses against the parental strain and the three VOC (Alpha, Beta, and Delta) that were assessed. Notably, even after three vaccinations with SpFN + AH, no cross-neutralizing antibodies against the Omicron variant were induced.

SpFN + ALFQ vaccine-induced significantly higher frequencies of S-2P protein-specific naive and S-2P protein-specific MBCs. In addition, a greater than twofold increase was observed in the frequencies of the S-2P protein-specific IgG1 expressing MBCs and the plasmablasts. This further translated into a fourfold increase in the amount of S-2P protein-specific IgG1 in the sera of mice vaccinated with SpFN + ALFQ compared to SpFN + AH. ELISpot analysis conducted with the splenocytes at week 6 also demonstrated a significantly higher number of S-2P protein-specific IgG and IgA spot-forming units in SpFN + ALFQ vaccinated mice. Antibody-secreting cells at week 6 indicated that these cells were not short-term plasmablasts, but rather LLPCs that were still present in the spleen.

Bone marrow analysis at week 21 demonstrated the superior ability of the SpFN + ALFQ vaccine to generate S-2P protein-specific plasma cells compared to SpFN + AH (2.0% vs. 0.06%). We postulate that the higher frequency of antigen-specific Tfh cells and GCs in the spleen and lymph nodes of SpFN + ALFQ vaccinated mice was associated with the induction of higher LLPCs in the bone marrow. Thus, the overall immune responses generated by SpFN + ALFQ vaccine were superior to that seen with SpFN + AH demonstrating the potency of the liposomal saponin-containing adjuvant ALFQ.

It has been documented that adjuvants play a critical role in the type of immune responses generated. Saponin adjuvants such as Matrix M, AS01B, and AS01E have previously been demonstrated to induce strong binding and neutralizing antibody responses with protective efficacy and a good example is the highly successful and efficacious shingles vaccine (Shingrix^®^) approved by the U.S. Food and Drug Administration that contains AS01B as the adjuvant^10,36–38^. Although, both AS01 and ALFQ are liposomes, the chemical composition and physical properties of the two formulations are markedly different. AS01 liposomes are uniform in size, ~100 nm, comprising an unsaturated phospholipid, dioleoyl phosphatidylcholine. In contrast, ALFQ contains two saturated phospholipids, DMPC and DMPG, and ranges in size from ~50 nm to as large as 30,000 nm in diameter. ALFQ contains 55 mole percent cholesterol versus 33.7% in AS01, and four times the amount of MPLA, and twice the amount of QS-21 than AS01. In addition, the two formulations also contain different types of MPLA as adjuvants. AS01 formulations contain native MPLA extracted from Salmonella minnesota R595 lipopolysaccharide (designated as MPL^®^), which consists of multiple natural congeners of MPLA, which have been reported to serve either as TLR4 agonists or antagonists in in vitro studies. In contrast, ALFQ contains only a single TLR4 agonist, 3D-PHAD^®^ (from Avanti Polar Lipids), a synthetic lipid A. These differences may translate into differences in adjuvanticity between the two formulations. Because of the polydispersity of ALFQ, the liposomes may be taken up by both macrophages and dendritic cells, whereas in the case of AS01 liposomes (50–100 nm), dendritic cells may serve as the predominant antigen-presenting cells. Thus, they would influence the functional characteristics of AS01 and ALFQ as vaccine adjuvants. Although, these two adjuvant formulations are similar in certain respects, yet they are distinct and unique.

The durability of the immune responses is a major challenge for the current COVID vaccines as the immune responses are not sustained over a period of time. In the present study, the binding antibody responses induced by SpFN + ALFQ were maintained until week 21, the final time point analyzed, compared to SpFN + AH group. It is therefore tempting to speculate that a boost with a protein antigen containing a potent adjuvant such as ALFQ might increase the durability of the immune responses generated by the current COVID vaccines.

Although, neutralizing antibody levels are highly predictive of protection against severe COVID-19^39^, binding antibodies with Fc-mediated effector functions can also contribute to protection against SARS-CoV-2^40–42^. SpFN + ALFQ vaccine displayed significantly higher ADCD and ADCP responses compared to SpFN + AH after both first and second vaccinations. However, we did not assess the Fc effector functions against other SARS-CoV-2 variants or a broad panel of sarbecoviruses at the early time points. Another limitation of the study is that it did not assess the breadth of the cellular and binding immune responses or conduct epitope mapping. Nonetheless, in summary, our data demonstrated that the SpFN + ALFQ vaccine promoted early induction of spike-specific IL-21 secreting Tfh cells, S-2P protein-specific naive, memory, and germinal center B cells resulting in the rapid induction of IgM and IgG binding antibodies that exhibited Fc effector functions and cross-neutralizing antibodies as early as day 7. These data thus emphasize that the early immune events induced by the SpFN + ALFQ vaccine are critical for the development of neutralization breadth and S-2P protein-specific long-lived plasma cells in the bone marrow. Future studies investigating SpFN + ALFQ-induced antigen-specific naive B cell repertoire and engagement of protective germline responses may provide insights in designing pan-coronavirus vaccines.

## Methods

### Study design

Female C57BL/6 mice (5–6 weeks of age) were obtained from The Jackson Laboratory. Mice were group housed and fed standard chow diets. C57BL/6 mice were housed in the animal facility of WRAIR, which is an American Association for Accreditation of Laboratory Animal Care-accredited facility, and cared for in accordance with local, state, federal, and institutional policies. Mice were vaccinated by intramuscular injection with SpFN + AH or SpFN + ALFQ. The number of mice at each time point varied from 5 to 10 for each group. The immunizations and experiments were repeated at least two times. Mice received either a single vaccination in the left hind leg quadriceps muscle or a second vaccination at week 3 in the right hind leg quadriceps muscle or a third vaccination at week 6 in the left hind leg quadriceps. Mice were euthanized on days 3, 5, 7, 10, 14, and 21 post-first vaccination or days 28, 35, 42 after the second vaccination or week 21 after all three vaccinations. Time point days 3, 7, and 28 and weeks 21 were repeated twice, each time with an *n* = 5 mice/time point/adjuvant or *n* = 10 for week 21. Time point days 5 and 10 were repeated three times. The 6-week time point with SpFN + ALFQ vaccine was repeated twice (*n* = 5, *n* = 9). Pre-vaccination peripheral blood was obtained on day −4. Blood was collected at the various time points and individual serum samples were stored at −80 °C. The dLNs and spleen were collected on days 3, 5, 7, and 10 following the first vaccination, and on week 4 (1 week after the second immunization). Spleens were also obtained at the weeks 6, and 21 after second and third vaccinations, respectively. Bone marrows were also collected at week 21.

### Ethics statement

Animal studies were carried out in accordance with the recommendations in the Guide for the Care and Use of Laboratory Animals of the National Institutes of Health. The protocols were approved by the Institutional Animal Care and Use Committee at the Walter Reed Army Institute of Research [Assurance number D16-00596 (A4117-01)]. Blood collection was performed as per WRAIR/NMRC VSP Guidelines on blood collection. Mice were euthanized using CO_2_ administered in a CO_2_ chamber, via regulated flow valve (in accordance with the most current 2020 AVMA Guidelines on Euthanasia), followed by cervical dislocation at the various time points mentioned in the study to collect spleen, lymph nodes, and bone marrow. Following euthanasia, mice were exsanguinated by cardiac puncture. Sample collection and processing are described below.

### Cell lines

P388D1 cells (ATCC, Manassas, VA, USA) were cultured in RPMI media (Quality Biologicals, Gaithersburg, MD, USA) supplemented with 2 mM L-glutamine (GE Lifesciences), 1% penicillin and streptomycin (GE Lifesciences), and 10% heat-inactivated FCS (Sigma) at 37 °C and 5% CO_2_. Cells were subcultured every 2–3 days. SARS-CoV-2 Spike-expressing expi293F cells were generated by transfection with linearized plasmid (pcDNA3.1) encoding codon-optimized full-length SARS-CoV-2 Spike protein matching the amino acid sequence of the IL-CDC-IL1/2020 isolate (GenBank ACC# MN988713). Stable transfectants were single-cell sorted and selected to obtain a high-level Spike surface expressing clone (293F-Spike-S2A).

### Immunogen-adjuvant formulation

The stabilized spike trimer-ferritin fusion recombinant protein nanoparticle (SpFN) was designed using the initial SARS-CoV-2 genome sequence from Wuhan, China (GenBank:MN9089473). The Helicobacter pylori ferritin molecule was linked to the C-terminal region of the pre-fusion stabilized ectodomain (residues 12-1158). The spike trimer formation on the ferritin molecule was stabilized by several modifications such as the introduction of two proline residues (K986P and V987P), a “GSAS” substitution at the furin cleavage site (residues 682-685), and changes in residues 1140-1161 to increase the coiled-coil interactions of the spike stalk region. The protein was transiently expressed as a soluble recombinant protein in mammalian Expi293 cells (Thermo Fisher Scientific). The self-assembled protein nanoparticle displaying the SARS-CoV-2 antigen on the nanoparticle surface was purified by lectin-affinity chromatography and size-exclusion chromatography. The purified spike protein ferritin nanoparticle (SpFN) was formulated in PBS with 5% glycerol at 1 mg/mL. The spike protein is displayed as a multivalent array (8 copies of the antigen) on the ferritin nanoparticle. The antigen, Spike protein-ferritin nanoparticle (SpFN) was provided by Dr. Gordon Joyce’s lab and the details of the immunogen design, purification, and characterization and formulation with Army Liposome Formulation (ALF) containing the saponin QS-21 (ALFQ) have been published^18,19^. SpFN was formulated in PBS with 5% glycerol at a concentration of 1 mg/ml and stored in aliquots at −80 °C until use. For vaccine preparations adjuvanted with ALFQ, lipids were mixed in a molar ratio of 9:1:12.2:0.114 (DMPC:DMPG:Chol: 3D-PHAD^®^), dried by rotary evaporation followed by overnight desiccation, rehydrated by adding Sorensen PBS, pH 6.2, followed by microfluidization to form small unilamellar vesicles (SUV) and filtration. QS-21 was added to SUV to form ALFQ (DMPC: DMPG: Chol: MPLA: QS-21; 9:1:12.2:0.114:0.044). The antigen was diluted in dPBS and mixed with 1.5X ALFQ liposomes containing 600 µg/ml 3D-PHAD and 300 µg /ml QS-21 to give a final concentration of 20 µg 3D-PHAD and 10 µg QS-21 per 50 µl dose^18,19^. Alternatively, SpFN antigen was adsorbed to Alhydrogel^®^. The stock solution of Alhydrogel^®^ (10 mg/ml aluminum, Al^3+^ Brenntag/Croda) was diluted to 900 µg/ml (1.5X) with DPBS and gently mixed with the appropriate volume and concentration of SpFN to give a final concentration of 30 µg Al^3+^ (AH) per 50 µl dose.

### Sample collection and processing

Inguinal and popliteal lymph nodes (LNs), spleen, and both tibias and femurs were harvested and placed in cold complete RPMI 1640 media (cRPMI) supplemented with 10% heat-inactivated Fetal Bovine Serum (Invitrogen, A3160401) 1% Pen-strep (Gibco, 15140122) and 1% L-glutamine (Gibco, 25030081). All tissues were kept on ice and processed immediately following collection.

Following collection, blood was centrifuged at 14,000 × *g* (maximum speed) for 30 min, 4 °C. Serum was collected and stored at −80 °C until use.

The inguinal and popliteal lymph nodes draining the vaccinated leg were processed and combined for analysis^19^. Briefly, mononuclear cells were obtained by homogenizing lymph nodes using the frosted ends of glass slides followed by centrifugation over Fico/Lite-LM (Atlanta Biologicals) density gradient. Mononuclear cells were then washed and resuspended in PBS with 2% FBS for flow cytometry analysis.

Mouse spleens were homogenized with the plunger of a 3-ml syringe and filtered through a 70μm cell strainer to obtain a single-cell suspension, followed by washing twice in PBS containing 2% FBS (FBS, Invitrogen). Cells were then counted using trypan blue exclusion, and used immediately for flow cytometry analysis or they were cryopreserved in freezing media (90% heat-inactivated FBS and 10% dimethyl sulfoxide) and stored in liquid nitrogen until use for the ELIspot assay.

BMs were flushed from both femurs and tibias from each mouse with cold PBS containing 2% FBS using a 1 mL 21 G × 1” syringe (BD Precisionglide^TM^, 305165) to obtain a single-cell suspension, followed by washing twice in PBS containing 2% FBS. Cells were then counted using trypan blue exclusion, cryopreserved in freezing media (90% heat-inactivated FBS and 10% dimethyl sulfoxide) and stored in liquid nitrogen until use.

### Enzyme-linked immunosorbent assay (ELISA) for IgM and IgG antibodies against RBD and S-2P proteins

The assay was performed as follows^18^. Briefly, each well of a 96-well Immulon “U” bottom plate was coated with 100 µl of either RBD or S-2P proteins (1 mg/ml) in Dulbecco’s PBS, pH 7.4 and stored overnight at 4 °C. Following blocking with buffer (PBS containing 0.5% milk and 0.1% Tween 20, pH 7.4), at room temperature (RT) for 2 h, individual serum samples were serially diluted twofold in blocking buffer and added to triplicate wells. The plates were incubated for 1 h at RT followed by the addition of secondary antibody specific to IgG or IgM (Horseradish peroxidase (HRP)-conjugated sheep anti-mouse IgG or goat anti-mouse IgM, gamma chain (The Binding Site; 1;1000; Cat.#AP272) or µ chain specific (Bethyl Laboratories, Inc.; 1:1000; Cat.#A90-101P), respectively. Color was developed by the addition of 2,20-Azinobis [3-ethylbenzothiazoline-6-sulfonic acid]-diammonium salt (ABTS) HRP substrate (KPL/Seracare) for 1 h at RT. The reaction was stopped by the addition of 1% SDS per well and the absorbance (A) was measured at 405 nm (A405) using an ELISA reader Spectramax (Molecular Devices, San Jose, CA) within 30 min of stopping the reaction. Antibody positive (anti-RBD mouse mAb; BEI resources) and negative controls were included on each plate. The results are expressed as end point titers, defined as the reciprocal dilution that gives an absorbance value that equals twice the background value (antigen-coated wells that did not contain the test sera, but had all other components added).

### Enzyme-linked immunosorbent assay (ELISA) for IgG1 antibodies against S-2P protein

Briefly, each well of a NUNC MaxiSorp 96-well flat bottom plate was coated with either 100 μl of S-2P protein or goat anti-mouse IgG Fab-UNLB standard (0.1 μg/well; Southern Biotech., Cat.#1015-01) in Dulbecco’s PBS and stored overnight at 4 °C. Next day, wells were blocked for 2 h at room temperature (RT) with blocking buffer (1× dPBS containing 0.5% milk and 0.1% Tween-20, pH 7.4), followed by the addition of twofold dilution of individual serum samples (triplicate) or mouse IgG1-UNLB standard (Southern Biotech., Cat.#0102-01) in triplicate in blocking buffer. After incubating for 1 h at RT, 100 μl of secondary antibody goat anti-mouse IgG1, human ads-HRP (1:1000; Southern Biotech., Cat.#1070-05) was added to each well followed by an additional hour of incubation at room temperature. The color was developed by the addition of a 1:1 mix of the two-component KPL ABTS peroxidase substrate system for 1 h at RT. The color development was stopped after 1 h by the addition 1% SDS stop solution. The absorbance was measured at 405 nm using a Spectramax plate reader. Positive and negative controls were included on each plate. The interpolated amount of S-2P protein-specific IgG1 antibody (μg/mL) in the serum was determined by using the IgG1 standard curve. The bar represents the average IgG1 concentration against the S-2P protein in the sera of mice vaccinated with SpFN + AH and SpFN + ALFQ at one week post second vaccination (week 4). The data are represented as bar graphs (Mean + SEM) and each dot represents the S-2P specific IgG1 concentration of the individual mouse (*n* = 6 per group).

### Surface plasmon resonance (Biacore)

Antibody avidity determinations were conducted using the Biacore 4000 surface plasmon resonance (SPR) system^43–45^. Briefly, the immobilizations were performed using a standard amine-coupling kit. The CM-5 sensor chip surface was activated with a 1:1 mixture of 0.4 M 1-ethyl-3-(3-dimethylaminopropyl) carbodiimide hydrochloride (EDC) and 0.1 M N-hydroxysuccinimide (NHS) for 600 s (GE Healthcare). Then S-2P (5 μg/mL and 10 μg/mL) and RBD (10 μg/mL and 20 μg/mL) proteins in 10 mM sodium acetate pH 4.5 were immobilized to spots 1, 2, 4, and 5 to each flow cell of the CM5 sensor chip resulting in 500–690 RU (low density) and 920–1043 RU (high density) for S-2P and 320–365 RU (low density) and 362–638 RU (high density) for RBD. Spot 3 in each flow cell was left unmodified to serve as a reference. The immobilized surface was then deactivated by 1.0 M ethanolamine-HCl pH 8.5 for 600 s. Following the surface preparation, heat-inactivated serum samples were diluted 1:50 in running buffer (10 mM Hepes, 300 mM NaCl, 0.005% Tween-20, pH7.4) were injected onto the antigen-immobilized surface for 250 s followed by dissociation for 900 sec. The bound surface was then enhanced with a 250-s injection of 30 μg/mL of goat anti-monkey IgG (secondary antibody). To regenerate the bound surface, 150 mM HCl was injected twice for 60 sec. Four replicates for each sample were collected at rate of 10 Hz, with an analysis temperature of 25 °C. All sample injections were conducted at a flow rate of 10 μL/min. Data analysis was performed using Biacore 4000 Evaluation software 4.1 with double subtractions for an unmodified surface and buffer for the blank. Fitting was conducted using the dissociation mode integrated with Evaluation software 4.1. The data are shown as Avidity score and this was calculated as Response Unit/K_d_.

### Flow cytometric phenotyping and intracellular cytokine staining (ICS)

Spectral flow cytometry was performed to analyze B and T cell subsets in the lymph nodes. Viable cells were identified with a LIVE/DEAD fixable blue dead cell stain kit (Invitrogen/ThermoFisher Scientific; Cat.#L34962; 1:200) and B and T cell phenotypes were analyzed using the fluorochrome-conjugated antibodies listed in Supplementary Table 1 and with the gating strategy shown in Supplementary Fig. 1a. Intracellular cytokine staining (ICS) was performed to identify SARS-CoV-2 spike (S)-specific T cells. Mononuclear cell suspension from the draining lymph nodes was plated at 0.5 × 10^6^ cells/well in a 96-well plate and stimulated with the spike peptide pools (JPT) at a final concentration of 1 μg/mL overnight at 37 °C in RPMI and 10% FBS. To prevent the secretion of cytokines, monensin (BioLegend) was added 1 h after the addition of peptides. Spike-specific T cells were measured by surface staining followed by fixation and permeabilization using eBioscience Foxp3/transcription factor fixation/permeabilization solution (eBioscience) and ICS (antibodies shown in Supplementary Table 2, gating strategy shown in Supplementary Fig. 1b). As negative and positive controls, cells were cultured in media without peptide stimulation or with phorbol 12-myristate 13-acetate (PMA) and ionomycin (BioLegend), respectively. T cells were considered to be responsive to peptide stimulation if the frequency of cytokine-expressing T cells was >0.01% of the parent population after background subtraction. Samples were analyzed on a 5-laser Cytek Aurora flow cytometer (Cytek Biosciences). The data were analyzed using FlowJo software v10.7.1 (BD Biosciences).

Approximately, 1 × 10^6^ fresh splenocytes were plated in a U-bottom plate and stimulated with SARS-CoV-2 spike protein peptide pool from JPT (S-2P, 1 μg/ml). The plates were incubated at 37 °C, 5% CO_2_ for 2 h followed by the addition of protein transport inhibitor (BD Golgi Plug™ containing Brefeldin A, 1 μg/mL, and BD Golgi Stop™ containing monensin, 1 μg/mL, BD Biosciences). The plates were incubated for an additional 14 h at 37 ^o^C, 5% CO_2_. For the positive control, cells were stimulated with ebiosciences stimulation cocktail containing PMA and Ionomycin (eBioscience™ Cell Stimulation Cocktail; 00-4970-03) while media served as a negative control. After the incubation period, cells were washed and stained with LIVE/DEAD Fixable Aqua Dead Cell Stain Kit (Invitrogen/ThermoFisher Scientific, 1:1000), followed by surface staining with a cocktail of fluorescently labeled antibodies specific for Tfh cells (Supplementary Table 3) for 30 min at 4 °C, washed twice with FACS buffer and then fixed/permeabilized for 40 min at 4 °C in the dark using the eBioscience™ Intracellular Fixation and Permeabilization Buffer Set (Thermo Fisher Scientific, 88-8824-00) as per the manufacturer’s instructions. Cells were then incubated with an intracellular antibodies cocktail (Supplementary Table 3) for 30 min at 4 °C, washed twice with permeabilization buffer, and resuspended in FACS buffer. Appropriate single-color compensation controls and Fluorescence control (FMO) were prepared simultaneously and were included in each analysis. Flow cytometry analysis was performed on a BD LSR II flow cytometer, and data were acquired using Diva software (BD Biosciences). The results were analyzed using FlowJo software version 10.7.1 (BD Biosciences). In each analysis, respective FMO controls were used to set up the gates or to identify the positive populations. The gating strategy applied for the evaluation of flow cytometry-acquired data is provided in Supplementary Fig. 2.

### Flow cytometry staining for SARS-CoV-2 spike-specific GC and memory B cells and plasma cells in spleen and bone marrow

SARS-CoV-2 spike protein (S-2P) was separately conjugated to either FITC or Alexa Fluor^®^647 using FITC Conjugation Kit (Fast)- Lightning-Link® (ab188285) or Alexa Fluor^®^647 Conjugation Kit (Fast)-Lightning-Link^®^ (ab269823), respectively, as per the manufacturer’s instructions. For staining antigen-specific GC B cells and memory B cells, 1 million fresh splenocytes (1 × 10^6^) were first incubated with Fc block (BD Biosciences, Cat.# 553142; 1:100) and LIVE/DEAD Fixable Aqua Dead Cell Stain Kit (Invitrogen/ThermoFisher Scientific, 1:1000) for 30 min, washed twice with FACS buffer, followed by surface staining with S-2P-FITC and S-2P-Alexa Fluor 647 and a cocktail of fluorescently labeled antibodies specific for B cell subsets (Supplementary Table 4) for 30 min at 4 °C, washed twice, and resuspended in FACS buffer. For staining antigen-specific plasma cells in the bone marrow, bone marrow cells (1 × 10^6^) were first incubated with Fc block and LIVE/DEAD Fixable Aqua Dead Cell Stain Kit (Invitrogen/ThermoFisher Scientific, 1:1000) for 30 min, washed twice with FACS buffer, followed by surface staining with a cocktail of fluorescently labeled antibodies specific for plasma cell subsets (Supplementary Table 5) for 30 min at 4 °C. After incubation, cells were washed twice with FACS buffer, fixed/permeabilized for 40 min at 4 °C in the dark using the eBioscience™ Intracellular Fixation and Permeabilization Buffer Set (Thermo Fisher Scientific, 88-8824-00) as per the manufacturer’s instructions. Cells were then incubated with S-2P-FITC and S-2P-Alexa Fluor 647 and intracellular antibodies (Supplementary Table 5) for 30 min at 4 °C, washed twice with permeabilization buffer, and resuspended in FACS buffer. Appropriate single-color compensation controls and Fluorescence control (FMO) were prepared simultaneously and were included in each analysis. Flow cytometric analysis was performed on a BD LSR II flow cytometer, and data were acquired using Diva software (BD Biosciences). The results were analyzed using FlowJo software version 10.7.1 (BD Biosciences). The gating strategy applied for the evaluation of flow cytometry-acquired data is provided in Supplementary Figs. 3–6.

### Immunohistochemistry/immunofluorescence

Inguinal and popliteal LN were harvested and fixed with 4% formalin at room temperature (RT). LN samples were collected at day 7 and week 4. Immunohistochemistry staining was conducted by Histowiz under a contract. The fixed lymph node sections were stained for CD3 and PD-1 for detecting Tfh and PNA and Ki67 for locating GC B cells. Immunohistochemistry was performed on a Bond Rx autostainer (Leica Biosystems) with heat-induced antigen retrieval by heating the slides in Bond Epitope Retrieval Solution 1 (ER1) (Leica Microsystems) using standard protocol. Slides were incubated with rabbit monoclonal CD3 primary antibody (abcam, ab16669, clone: SP7, 1:100), rabbit polyclonal Ki67 primary antibody (abcam, ab15580, 1:800), rabbit monoclonal PD1 primary antibody (abcam, ab214421, clone: EPR20665, 1:500), PNA-Biotin primary antibody (Vector Laboratories, B-1075, 1:1500) and Streptavidin-HRP tertiary (Leica, RE7104-CE, ready-to-use). Bond Polymer Refine Detection (Leica Biosystems) was used according to the manufacturer’s protocol. After staining, sections were dehydrated and film coverslipped using a TissueTek-Prisma and Coverslipper (Sakura). Whole slide scanning (40×) was performed on an Aperio AT2 (Leica Biosystems).

### SARS-CoV-2 pseudovirus neutralization assay

The construction of the various plasmids and the neutralization assay have been described in detail^18^. The S expression plasmid sequences for SARS-CoV-2 [Wuhan1, B.1.1.7 (Alpha), B.1.351 (Beta), B.1.617.2 (Delta), and B.1.1.529/BA.1 (Omicron)] were codon optimized and modified to remove an 18 amino acid endoplasmic reticulum retention signal in the cytoplasmic tail, which allowed increased S incorporation into pseudovirions (PSV) and thereby improved infectivity. Virions pseudotyped with the vesicular stomatitis virus (VSV) G protein were used as a nonspecific control. SARS-CoV-2 pseudovirions (PSV) were produced by co-transfection of HEK293T/17 cells with a SARS-CoV-2 S plasmid (pcDNA3.4) and HIV-1 NL4-3 luciferase reporter plasmid (The reagent was obtained through the NIH HIV Reagent Program, Division of AIDS, NIAID, NIH: Human Immunodeficiency Virus 1 (HIV-1) NL4-3 DEnv Vpr Luciferase Reporter Vector (pNL4- 3.Luc.R-E-), ARP-3418, contributed by Dr. Nathaniel Landau and Aaron Diamond). The SARS-CoV-2 S expression plasmid sequence was derived from the Wuhan seafood market pneumonia virus isolate Wuhan-Hu-1, complete genome (GenBank: MN908947). Infectivity and neutralization titers were determined using ACE2-expressing HEK293 target cells (Integral Molecular) in a semiautomated assay format using robotic liquid handling (Biomek NXp Beckman Coulter). Test sera were diluted 1:40 in growth medium and 3-fold serially diluted; then 25 mL/well was added to a white 96-well plate followed by the addition of an equal volume of diluted SARS-CoV-2 PSV to each well. The plates were incubated for 1 h at 37 °C. Target cells were added to each well (40,000 cells/well) and plates were incubated for an additional 48 h in a CO_2_ incubator at 37 °C. RLUs were measured with the EnVision Multimode Plate Reader (Perkin Elmer, Waltham, MA) using the Bright-Glo Luciferase Assay System (Promega Corporation, Madison, WI). Neutralization dose–response curves were fitted by nonlinear regression with a five-parameter curve fit using the LabKey Server and the final titers are reported as the reciprocal of the dilution of serum necessary to achieve 50% neutralization (ID50, 50% inhibitory dilution) and 80% neutralization (ID80, 80% inhibitory dilution). The horizontal black dotted lines in the Figures represent the lower limit of detection for the assay. Assay equivalency for SARS-CoV-2 was established by participation in the SARS-CoV-2 Neutralizing Assay Concordance Survey (SNACS) run by the Virology Quality Assurance Program and External Quality Assurance Program Oversite Laboratory (EQAPOL) at the Duke Human Vaccine Institute, sponsored through programs supported by the National Institute of Allergy and Infectious Diseases, Division of AIDS.

### ELISpot

Multiscreen PVDF plates (Millipore) were separated into three sections and coated with anti-IgG/anti-IgA antibody [1 μg/mL; ImmunoSpot, Cellular Technology Limited (C.T.L.)], S-2P protein (10 μg/mL) and RBD protein (10 μg/mL) and placed in a humid chamber overnight at 4 °C. The following day, the capture/antigen solution was decanted, the plates were washed with sterile phosphate buffered saline, 1× (PBS) followed by the addition of sterile assay media (RPMI with 10% FBS, Pen/Strep, L-glutamine, nonessential amino acids, sodium pyruvate, HEPES, and 2-mercaptoethanol) to block for 1 h at RT. Following the blocking, splenocytes from week 6 that had been stimulated with B-Poly-S™ reagent (C.T.L.; 1:1000) for four days and kept in a CO_2_ incubator at 37 °C were added to the S-2P and RBD-coated wells plates (100,000 cells/well for S2P and 400,000 cells/well for RBD wells), the plates were then placed in the CO_2_ incubator at 37 °C or 8 h. The plates were washed twice with PBS containing 0.05% Tween20 (PBS-T) then, anti-mouse IgG/IgA detection solution (C.T.L.) was added, and incubated for 2 h at RT. The plates were washed three times with PBS-T followed by the addition of tertiary solution (C.T.L.) for 1 h at RT. The plates were developed with the CTL-TrueBlue substrate solution (C.T.L.) for 10 min at RT, washed twice with distilled water followed by the CTL-TrueRed substrate solution (C.T.L.) for 7 min at RT and rinsed gently with tap water. The plates were allowed to dry completely. Counting and data analysis was conducted using the AID Autoimmun Diagnostica GmbH ELISpot reader and software. B cell responses were considered positive when the mean spot count exceeded the mean ± 3 SD of the negative control wells.

### Fc effector function assays

The Antibody-Dependent Cellular Phagocytosis **(**ADCP) assay was adapted from Ackerman et al.^46^. Briefly, biotinylated SARS-CoV-2 Spike trimer (Hexapro) was incubated with yellow-green streptavidin-fluorescent beads (Molecular Probes) for 2 h at 37 °C. 10 μL of a 1:100-fold dilution of beads-protein was incubated 2 h at 37 °C with 100 μL of 900-fold dilutions of serum samples before addition of 20,000 P388D1 cells per well. After 18 h incubation at 37 °C, the cells were fixed with 2% formaldehyde solution (Tousimis, Rockville MD USA) and fluorescence was evaluated on a LSRII (BD Bioscience, San Jose, CA, USA). The phagocytic score was calculated by multiplying the percentage of bead-positive cells by the geometric mean fluorescence intensity of the bead-positive cells and dividing by 10^4^.

The Antibody Dependent Complement Deposition (ADCD) assay was adapted from Fischinger et al.^47^. Briefly, 293F-Spike-S2A cells were incubated with 100 μL of serum diluted 10-fold in R10 media for 30 min at 37 °C. Lyophilized guinea pig complement (CL4051, Cedarlane, Burlington, Canada) was reconstituted per the manufacturer’s instructions in 1 mL cold water and centrifuged to remove aggregates for 5 min at 4 °C. Cells were washed with PBS and resuspended in 200 μL of guinea pig complement, which was prepared at a 1:50 dilution in Gelatin Veronal Buffer with Ca^2+^ and Mg^2+^ (IBB-300×, Boston BioProducts, Ashland, MA). After incubation at 37 °C for 20 min, cells were washed in PBS 15 mM EDTA (ThermoFisher Scientific) and stained with an anti-guinea pig complement C3 FITC (polyclonal, ThermoFisher Scientific, Cat# PA1-28933; 1:100). Cells were fixed with 4% formaldehyde solution and fluorescence was evaluated on a LSRII (BD Bioscience).

### Statistical analyses

The Figure legends detail all quantification and statistical analyses, inclusive of animal numbers (*n*), and statistical tests. Serum IgG endpoint titers and pseudovirus neutralizing antibody titers are depicted as log (10) transformed values prior to statistical analyses. For mouse sera ELISA binding antibodies, SARS-CoV-2 pseudovirus neutralization ID50 and ID80 values, and avidity comparisons between the two vaccine groups were assessed with an unpaired two-tailed Mann–Whitney *U* tests. Fc effector function assay comparisons between SpFN formulated with ALFQ and AH were assessed using an unpaired two-tailed Mann–Whitney *U* tests. *P* values < 0.05 were considered as significant and are shown within the Figs. Flow cytometric data were analyzed using FlowJo v.10.7.1 (BD Biosciences). Data were displayed as dot plots or bar graphs. Correlation analysis was determined by Spearman correlation. All the graphs were plotted, and the statistical analyses were conducted using GraphPad Prism v.9.0.2.

### Reporting summary

Further information on research design is available in the Nature Research Reporting Summary linked to this article.

## Supplementary information

Supplementary Information

REPORTING SUMMARY

## Data Availability

All reagents will be made available on request after completion of a Materials Transfer Agreement. All data supporting the findings of this study are found within the paper and its supplemental information. Any additional information required to reanalyze the data reported in this paper is available from the Lead Contact author upon request.
