# Supplementary material for: SARS-CoV-2 spike-ferritin-nanoparticle adjuvanted with ALFQ induces long-lived plasma cells and cross-neutralizing antibodies
Source: NPJ Vaccines. 2023 Mar 18;8:43. doi: 10.1038/s41541-023-00638-6 (PMC10024299; doi:10.1038/s41541-023-00638-6)
Supplement: Supplementary file 1 — Supplementary Information [file 41541_2023_638_MOESM1_ESM.pdf]

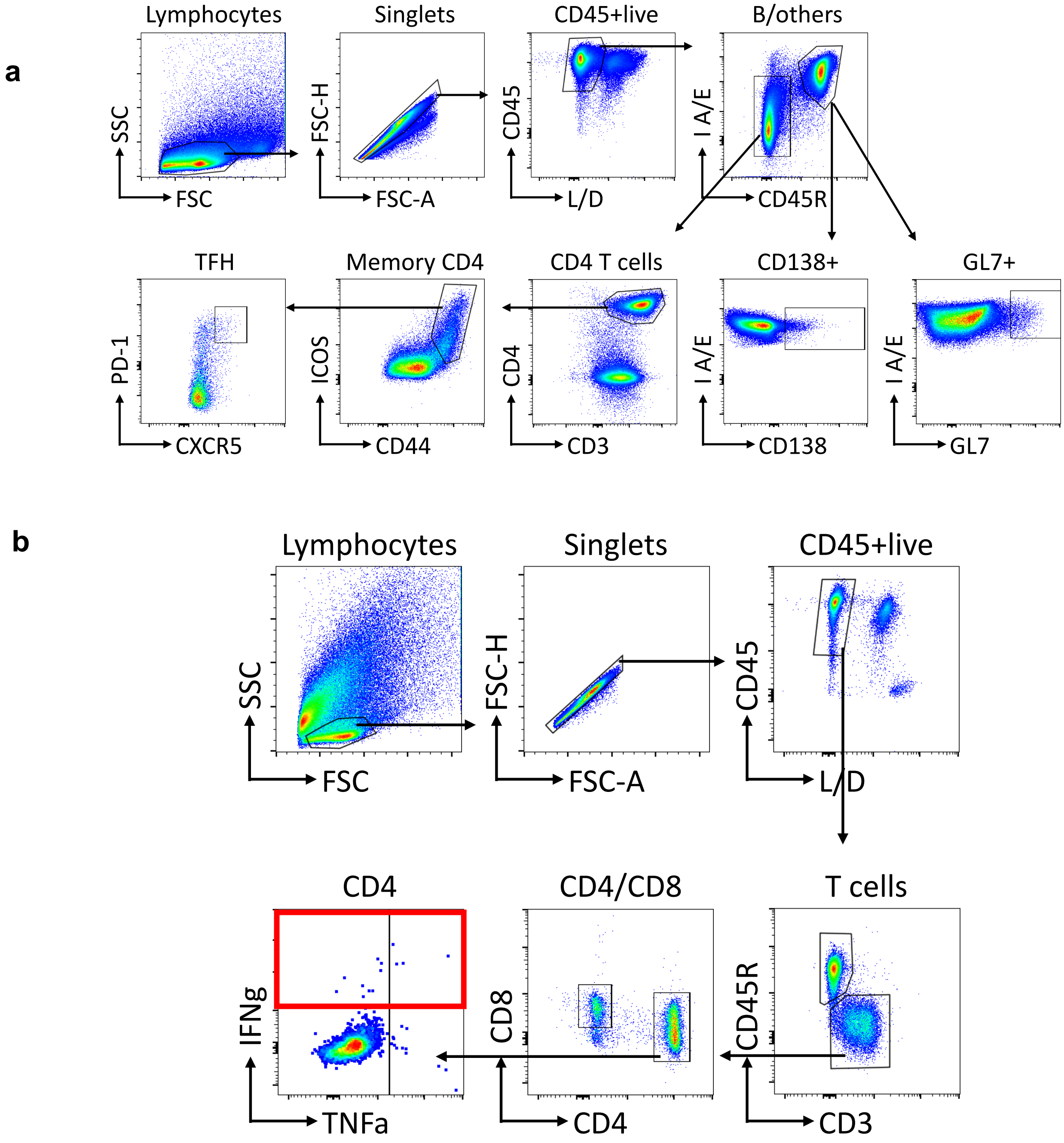


**Supplementary Figure 1.** Flow gating strategies for the various cell subsets in the draining lymph node of mice. (**a**) Gating strategy for T follicular helper cells (Tfh), plasmablasts, and germinal center (GC) B cells (**b**) Gating strategy for Spike-specific IFN-γ $^{+}$ CD4$^{+}$ T cells.

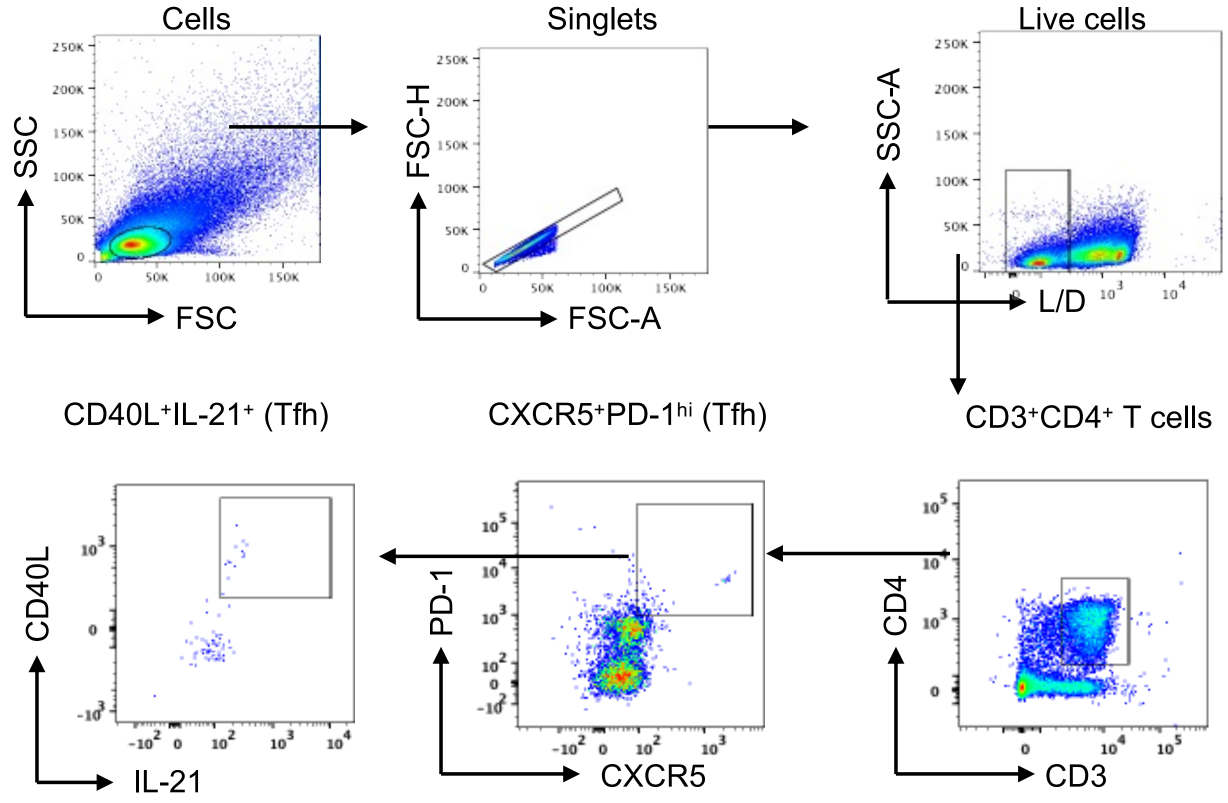


**Supplementary Figure 2.** Flow gating strategy for T follicular helper cells (Tfh) cells and Spike-specific IL21⁺ Tfh cells in the spleen of mice.

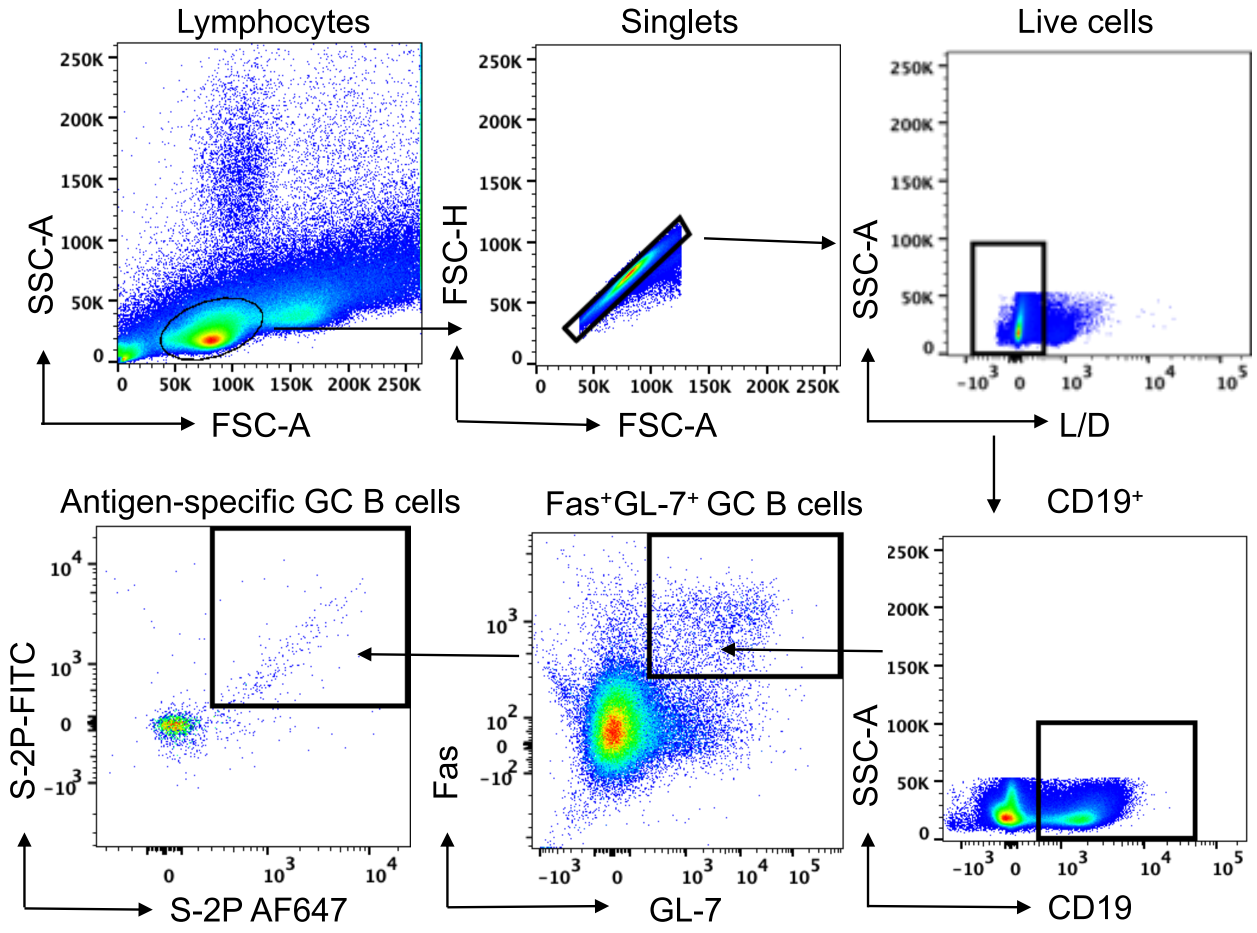


**Supplementary Figure 3.** Flow gating strategy for germinal center (GC) B cells and S-2P protein-specific GC B cells in the spleen of mice.

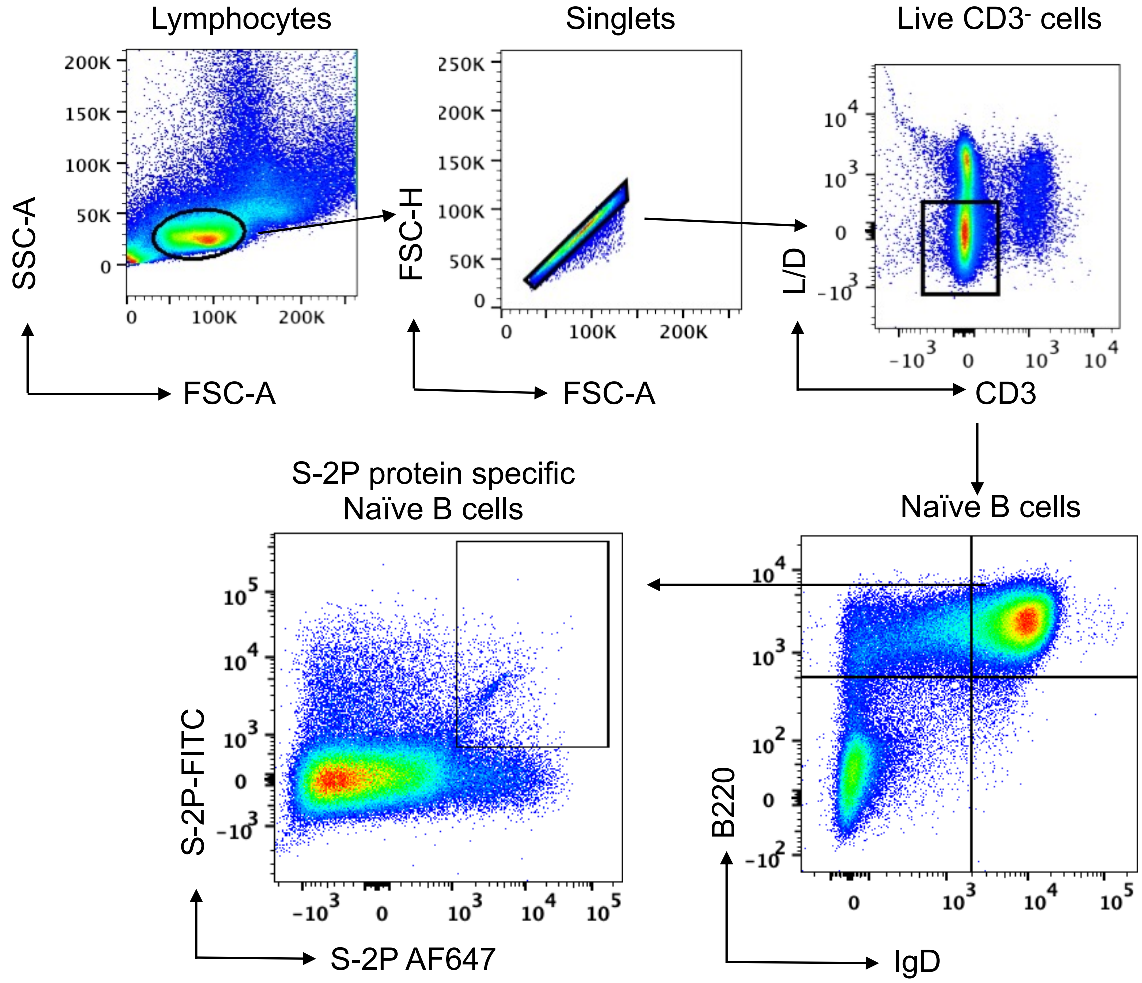


**Supplementary Figure 4.** Flow gating strategy for naïve B cells and S-2P protein-specific naive B cells in the spleen of mice.

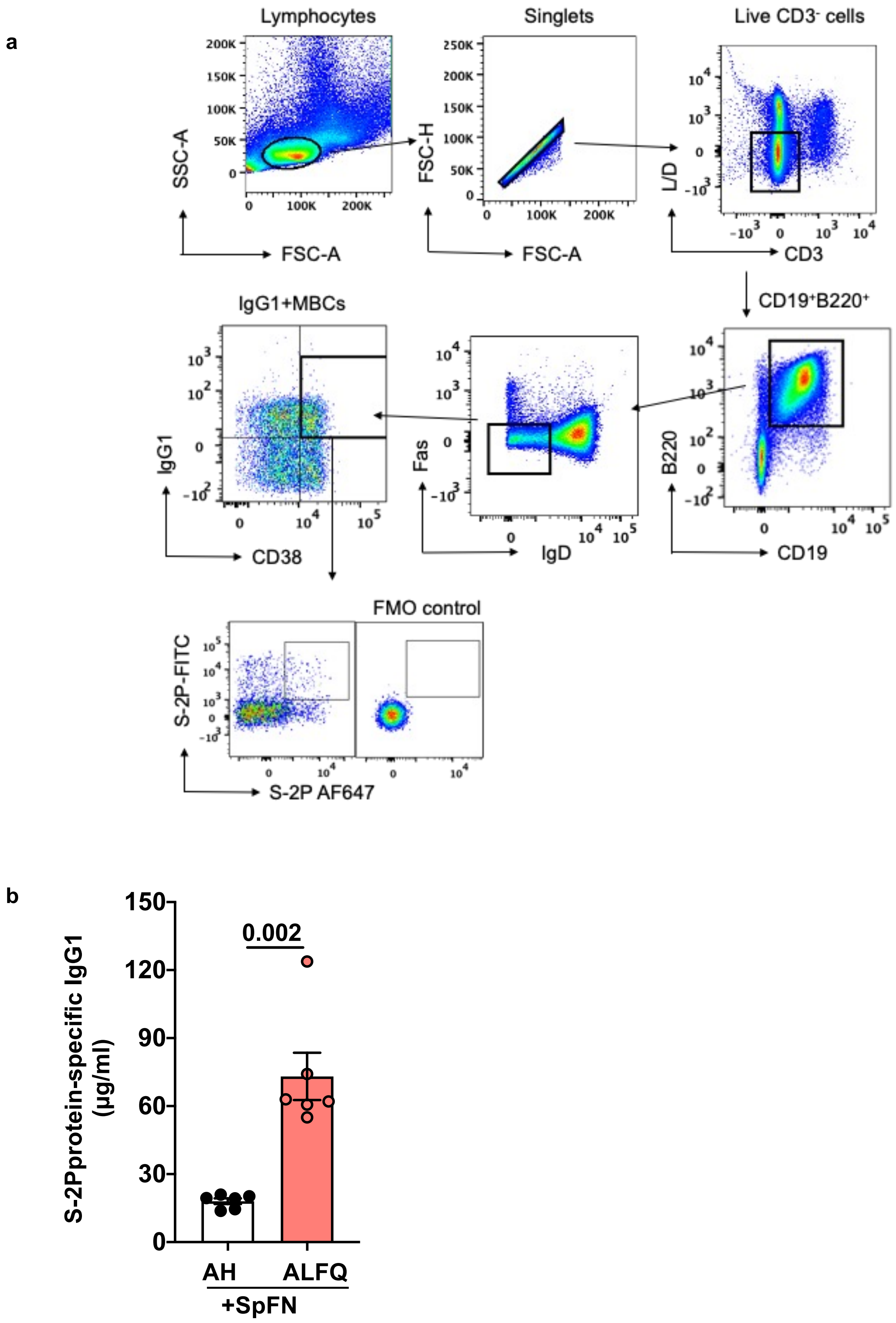


**Supplementary Figure 5 (a)** Flow gating strategy for IgG1$^{+}$Memory B cells and S-2P protein-specific IgG1$^{+}$ MBCs in the spleen of mice **(b)** IgG1 titers against the S-2P protein in the sera of mice vaccinated with SpFN+AH and SpFN+ALFQ at one week post second vaccination (week 4).

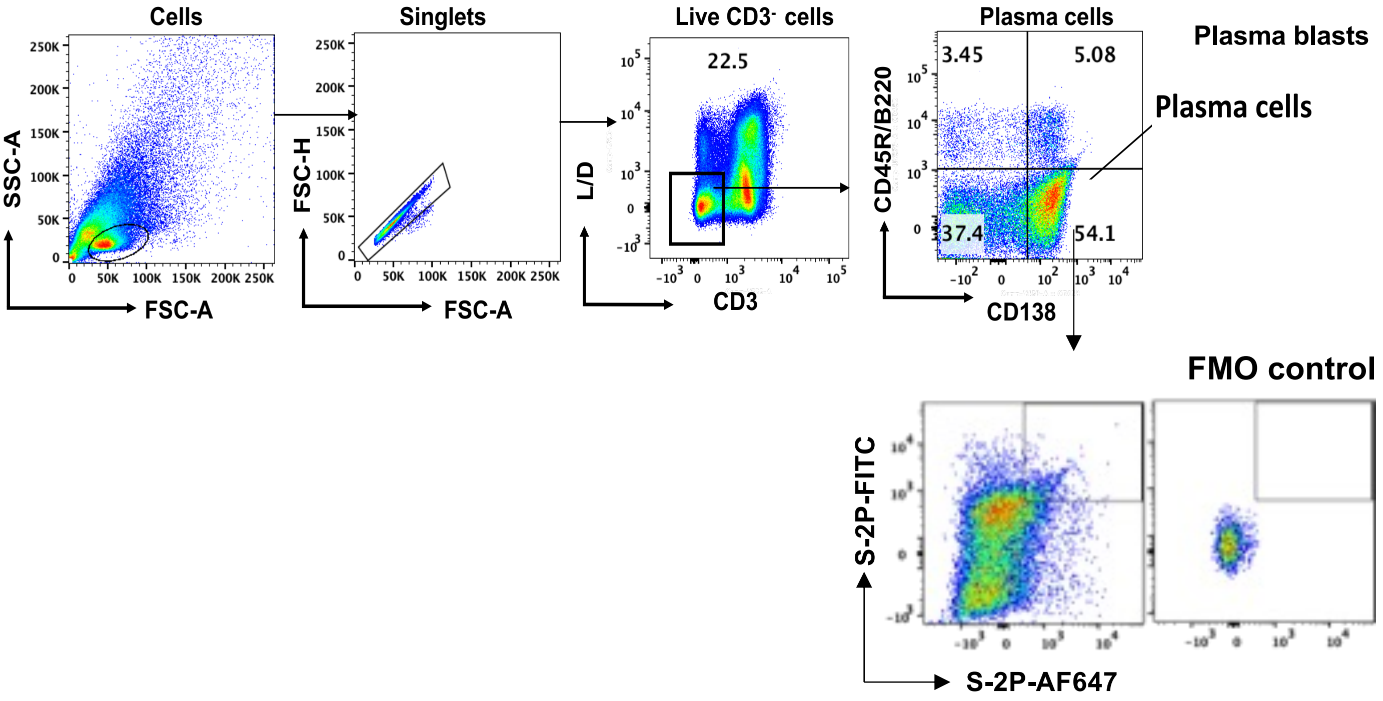


**Supplementary Figure 6.** Representative flow gating strategy for plasmablasts in the spleen and plasma cells and intracellular S-2P protein-specific long lived plasma cells (LLPCs) in the bone marrow of mice.

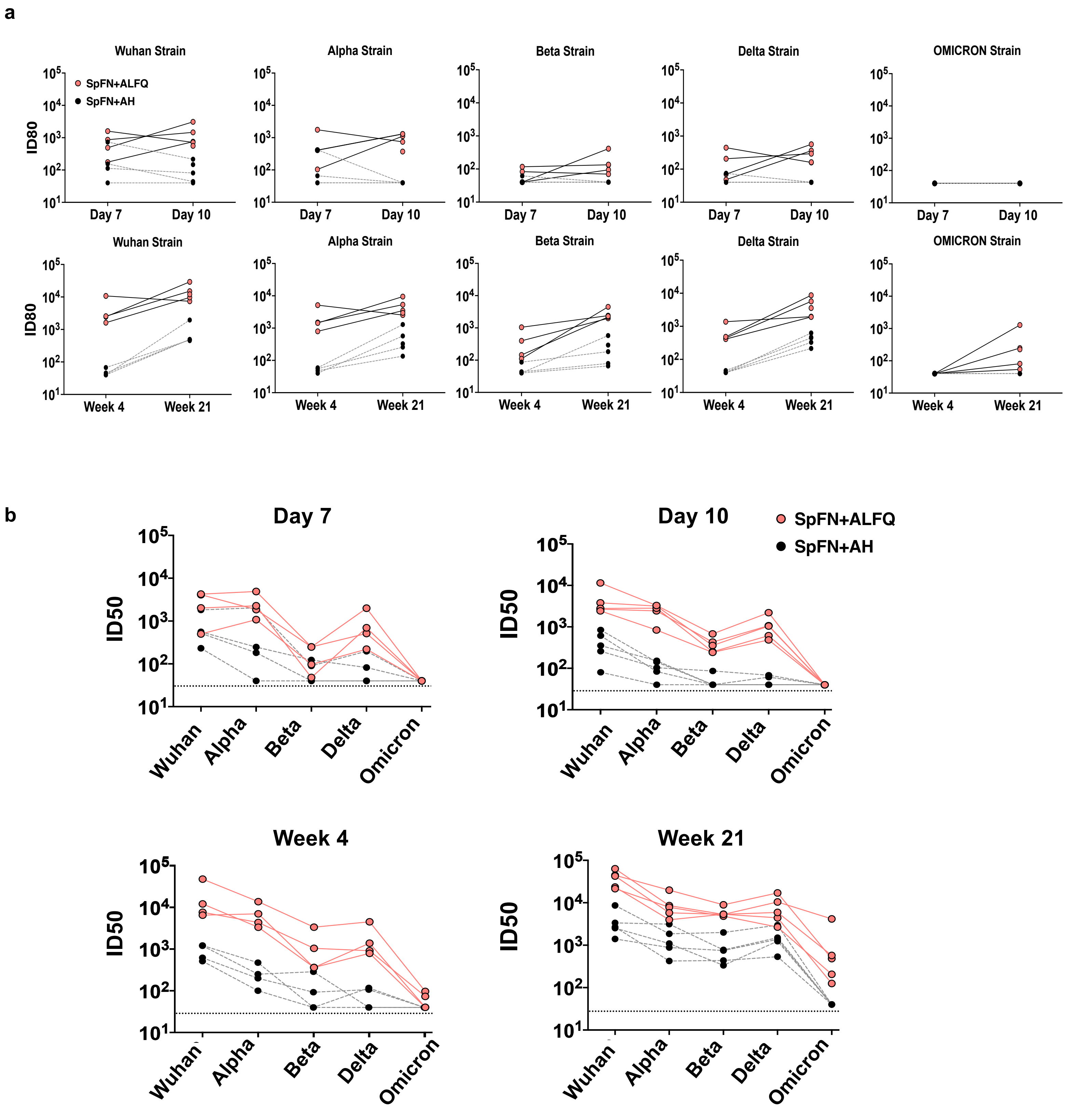


**Supplementary Figure 7. (a)** Pseudovirus neutralization titers (ID80) for Wuhan and individual variants of concern (VOC; Alpha, Beta, Delta, and Omicron) for each mouse on days 7 and 10, and on weeks 4 and 21 are shown.**(b)** Pseudovirus neutralization titers (ID50) against Wuhan and variants of concern (VOC; Alpha, Beta, Delta, and Omicron) on days 7, 10, weeks 4, and 21.

**Supplementary Tables**

**Supplementary Table 1**

| **Fluorochrome** | **Antigen** | **Clone** | **Manufacturer** | **Cat#** | **Dilution Used** |
|---|---|---|---|---|---|
| | LIVE/DEAD™ Fixable Blue Dead Cell Stain Kit | All species | ThermoFisher Scientific | L34962 | 1:200 |
| BUV395 | CD45R | RA3-6B2 | BD | 563793 | 1:100 |
| BUV661 | CD69 | H1.2F3 | BD | 741478 | 1:20 |
| BUV737 | CD3e | 145-2C11 | BD | 612771 | 1:100 |
| BV421 | CD138 | 281-2 | BioLegend | 142508 | 1:30 |
| BV510 | CD44 | IM7 | BioLegend | 103044 | 1:200 |
| BV570 | CD62L | MEL-14 | BioLegend | 104433 | 1:200 |
| BV650 | ICOS | C398.4A | BioLegend | 313550 | 1:60 |
| BV711 | I A/E | M5/114.15.2 | BioLegend | 107643 | 1:200 |
| FITC | CXCR5 | L138D7 | BioLegend | 145520 | 1:100 |
| PerCP-Cy5.5 | CD24 | M1/69 | BioLegend | 101824 | 1:200 |
| PE-Dazzle594 | CD4 | GK1.5 | BioLegend | 100456 | 1:80 |
| PE-Cy5 | CCR7 | 4B12 | BioLegend | 120114 | 1:20 |
| PE-Cy7 | GL7 | GL7 | BioLegend | 144620 | 1:100 |
| APC | PD1 | 29F.1A12 | BioLegend | 135210 | 1:50 |
| APC-Cy7 | CD19 | 6D5 | BioLegend | 115530 | 1:60 |
| APC-Fire810 | CD45 | 30-F11 | BioLegend | 103174 | 1:600 |

**Supplementary Table 2**

| **Fluorochrome** | **Antigen** | **Clone** | **Manufacturer** | **Cat#** | **Dilution Used** |
|---|---|---|---|---|---|
| **Surface** | | | | | |
| | LIVE/DEAD™ Fixable Blue Dead Cell Stain Kit | All species | ThermoFisher Scientific | L34962 | 1:200 |
| BUV395 | CD3e | 145-2C11 | BD | 563565 | 1:100 |
| BV510 | CD44 | IM7 | BioLegend | 103044 | 1:200 |
| BV570 | CD62L | MEL-14 | BioLegend | 104433 | 1:200 |
| BV605 | CD69 | H1.2F3 | BioLegend | 104530 | 1:20 |
| BV650 | ICOS | C398.4A | BioLegend | 313550 | 1:60 |
| FITC | CXCR5 | L138D7 | BioLegend | 145520 | 1:100 |
| PerCP-Cy5.5 | CD8 | 53-6.7 | BioLegend | 100734 | 1:200 |
| PE-Dazzle594 | CD4 | GK1.5 | BioLegend | 100456 | 1:600 |

| | | | | | |
|---|---|---|---|---|---|
| PE-Cy5 | CCR7 | 4B12 | BioLegend | 120114 | 1:20 |
| APC | PD1 | 29F.1A12 | BioLegend | 135210 | 1:50 |
| APC-Cy7 | CD19 | 6D5 | BioLegend | 115530 | 1:100 |
| APC-Fire810 | CD45 | 30-F11 | BioLegend | 103174 | 1:600 |
| **Intracellular** | | | | | |
| eFluor 450 | IL21 | FFA21 | eBioscience | 48-7211-82 | 1:50 |
| BV480 | Ki67 | B56 | BD | 566109 | 1:50 |
| BV711 | IFN-γ | XMG1.2 | BioLegend | 505836 | 1:80 |
| BV785 | TNF-α | MP6-XT22 | BioLegend | 506341 | 1:200 |

**Supplementary Table 3**

| Fluorochrome | Antigen | Clone | Manufacturer | Cat# | Dilution Used |
|---|---|---|---|---|---|
| **Surface** | | | | | |
| BV510 | Live/Dead Fixable Aqua Dead Cell Stain Kit | All species | ThermoFisher Scientific | L34966 | 1:1000 |
| BUV737 | CD3e | 145-2C11 | BD Biosciences | 612771 | 1:200 |
| BUV395 | CD4 | GK1.5 | BD Biosciences | 565974 | 1:100 |
| BV711 | CD8 | 53-6.7 | BD Biosciences | 563046 | 1:80 |
| APC-H7 | CD45RA | RA3-6B2 | BD Biosciences | 565371 | 1:100 |
| BV785 | CCR7 | 4B12 | BioLegend | 120127 | 1:80 |
| PE-eFluor610 | CXCR5 | SPRCL5 | eBioscience | 61-7185-82 | 1:40 |
| PE-Cy7 | PD1 | J43 | eBioscience | 25-9985-82 | 1:40 |
| BV650 | CD69 | 809220 | BD Biosciences | 748098 | 1:50 |
| **Intracellular** | | | | | |
| V450 | IFN-γ | XMG1.2 | BD Biosciences | 560661 | 1:20 |
| BV605 | CD154 | MR1 | BD Biosciences | 745242 | 1:20 |
| FITC | TNF-α | MP6-XT22 | BD Biosciences | 554418 | 1:40 |
| PerCP-Cy5 | IL4 | 11B11 | BD Biosciences | 560700 | 1:20 |
| PE | IL2 | 3C7 | BD Biosciences | 553075 | 1:40 |
| APC | IL21 | FFA21 | eBioscience | 17-7211-82 | 1:20 |

**Supplementary Table 4**

| Fluorochrome | Antigen | Clone | Manufacturer | Cat# | Dilution Used |
|---|---|---|---|---|---|
| Purified | CD16/CD32 | 2.4G2 | BD Biosciences | 553142 | 1:100 |

| | | | | | |
|---|---|---|---|---|---|
| BV510 | Live/Dead Fixable Aqua Dead Cell Stain Kit | All species | ThermoFisher Scientific | L34966 | 1:1000 |
| BUV395 | CD45R (B220) | RA3-6B2 | BD Biosciences | 563793 | 1:100 |
| BB700 | CD19 | 1D3 | BD Biosciences | 566411 | 1:100 |
| PE | IgD | 11-26c.2a | Biolegend | 405706 | 1:100 |
| Brilliant Violet 711 | IgM | RMM-1 | Sony | 2632695 | 1:100 |
| Brilliant Violet 605 | IgG1 | A85-1 | BD Biosciences | 563285 | 1:50 |
| PE-Cy7 | GL7 | GL7 | Biolegend | 144620 | 1:100 |
| Brilliant Violet 421 | CD138 | 281-2 | Biolegend | 142508 | 1:30 |
| PE-Cy5 | CD38 | 90 | Abcam | ab25043 | 1:50 |
| Brilliant Violet 650 | CD27 | LG.3A10 | Biolegend | 124233 | 1:50 |
| APC-Cy7 | CD3 | 17A2 | Sony | 1101110 | 1:200 |
| PE-CF594 | CD95 | Jo2 | BD Biosciences | 562499 | 1:50 |
| FITC | S-2P protein (Joyce et al., 2020) | Lightning-Link® kit | Abcam | ab188285 | 1:20 |
| Alexa Fluor 647 | S-2P protein (Joyce et al., 2020) | Lightning-Link® kit | Abcam | ab269823 | 1:20 |

**Supplementary Table 5**

| Fluorochrome | Antigen | Clone | Manufacturer | Cat# | Dilution Used |
|---|---|---|---|---|---|
| **Surface** | | | | | |
| Purified | CD16/CD32 | 2.4G2 | BD Biosciences | 553142 | 1:100 |
| BV510 | Live/Dead Fixable Aqua Dead Cell Stain Kit | All species | ThermoFisher Scientific | L34966 | 1:1000 |
| BUV395 | CD45R (B220) | RA3-6B2 | BD Biosciences | 563793 | 1:100 |
| BB700 | CD19 | 1D3 | BD Biosciences | 566411 | 1:100 |
| Brilliant Violet 421 | CD138 | 281-2 | Biolegend | 142508 | 1:30 |
| APC-Cy7 | CD3 | 17A2 | Sony | 1101110 | 1:200 |
| **Intracellular** | | | | | |
| FITC | S-2P protein (Joyce et al., 2020) | Lightning-Link® kit | Abcam | ab188285 | 1:20 |
| Alexa Fluor 647 | S-2P protein (Joyce et al., 2020) | Lightning-Link® kit | Abcam | ab269823 | 1:20 |

**KEY RESOURCES TABLE**

| **Reagent or Resource** | **Source** | **Identifier** | **Dilution Used** |
|---|---|---|---|
| **Antibodies** | | | |
| Anti-RBD mouse mAb, 240C | BEI Resources | NR-616 | 1μg/mL |
| Horseradish peroxidase (HRP)-conjugated sheep anti-mouse IgG, gamma chain specific | The Binding Site | Cat# AP272 | 1:1000 |
| Horseradish peroxidase (HRP)-conjugated goat anti-Mouse IgM Antibody | Bethyl labs | Cat#A90-101P | 1:1000 |
| Peroxidase substrate A | Seracare | Cat#5120-0035 | neat |
| Peroxidase substrate B | Seracare | Cat#5120-0038 | neat |
| Goat Anti-Mouse IgG1, Human ads-HRP | SouthernBiotech | Cat#1070-05 | 1:1000 |
| Mouse IgG1-UNLB (15H6) | SouthernBiotech | Cat# 0102-01 | 0.03-250ng/mL |
| Goat Anti-Mouse IgG Fab-UNLB | SouthernBiotech | Cat# 1015-01 | 0.1 μg/well |
| Rabbit monoclonal CD3 primary antibody, clone: SP7 | Abcam | Cat# ab16669 | 1:100 |
| Rabbit polyclonal Ki67 primary antibody | Abcam | Cat# ab15580 | 1:800 |
| Rabbit monoclonal PD1 primary antibody, clone: EPR20665 | Abcam | Cat# ab214421 | 1:500 |
| PNA-Biotin primary antibody | Vector Laboratories | Cat# B-1075 | 1:1500 |
| Streptavidin-HRP tertiary | Leica | Cat# RE7104-CE | Ready to use |
| Bond Polymer Refine Detection | Leica Biosystems | N/A | N/A |
| Mouse IgG/IgA Double-Color ELISPOT kit | ImmunoSpot®, Cellular Technology Limited (C.T.L.) | N/A | N/A |
| **Chemicals, peptides and recombinant proteins** | | | |

| | | | |
|---|---|---|---|
| 2,20-Azinobis [3ethylbenzothiazoline-6-sulfonic acid]-diammonium salt (ABTS) | KPL/Seracare | Cat# 5120-0034 (50-64-00) | neat |
| SARS-CoV-2 S-2P protein | Joyce et al., 2020) | N/A | 0.1µg/well |
| SARS-CoV-2 RBD protein | (*48*) | N/A | 0.1µg/well |
| ALFQ | This manuscript | N/A | 20 µg 3D-PHAD; 10 µg QS-21/50 µl dose/mouse |
| DPBS | Quality Biologicals | Cat# 114-057-101 | N/A |
| Bovine serum albumin | Sigma-Aldrich | Cat# A8327 | N/A |
| FreeStyle 293 Expression Medium | GIBCO | Cat# 12338002 | N/A |
| Alhydrogel 2% | Croda inc. | Cat# AJV3012/0250/VP01 | 30µg$Al^{3+}$ (AH) /50µl dose/mouse |
| Tween 20 | Sigm-Aldrich | Cat#P1379 | 0.1% (ELISA) 0.005% (Biacore) |
| 10% SDS | Invitrogen | Cat# 24730-020 | 1:10 |
| Gelatin Veronal Buffer with $Ca^{2+}$ and $Mg^{2+}$ | Boston BioProducts | Cat# IBB-300x | N/A |
| Powdered milk | Giant | Cat#A614-1005 | 0.5% |
| HIV-1 NL4-3 luciferase reporter plasmid | NIH HIV Reagent Program | ARP-3418 | N/A |
| Anti-guinea pig complement C3 FITC (polyclonal) | ThermoFisher Scientific | Cat# PA1-28933 | 1:100 |
| Lyophilized guinea pig complement | Cedarlane | Cat# CL4051 | 1:50 |
| FITC Conjugation Kit (Fast)- Lightning-Link® | Abcam | Cat# ab188285 | N/A |
| Alexa Fluor®647 Conjugation Kit (Fast)- Lightning-Link® | Abcam | Cat# ab269823 | N/A |
| **Experimental models: Cell lines** | | | |

| | | | |
|---|---|---|---|
| Freestyle 293F cells | ThermoFisher Scientific | Cat# R7007 | N/A |
| Expi293F cells | ThermoFisher Scientific | Cat# A14635 | N/A |
| P388D1 cells | ATCC | Cat#CCL-46 | N/A |
| ACE2-expressing HEK293 target cells | Integral Molecular | N/A | N/A |
| HEK293T/17 cells | ATCC | Cat# CRL-11268 | N/A |
| **Experimental models: strains** | | | |
| Mus musculus, strain C57BL/6J | Jackson Laboratories | JAX stock #000664 | N/A |
| **Software** | | | |
| GraphPad Prism | GraphPad Software, LLC | Version 9.0.2 | N/A |
| FlowJo software | BD Biosciences | Version 10.7.1 | N/A |
| Biacore 4000 Evaluation software | GE Healthcare Life Sciences/Cytiva | Version 4.1 | N/A |
| GmbH ELISpot reader and software | AID Autoimmun Diagnostica | N/A | N/A |
