# Supplementary material for: SARS-CoV-2 spike-ferritin-nanoparticle adjuvanted with ALFQ induces long-lived plasma cells and cross-neutralizing antibodies
Source: NPJ Vaccines. 2023 Mar 18;8:43. doi: 10.1038/s41541-023-00638-6 (PMC10024299; doi:10.1038/s41541-023-00638-6)
Supplement: Supplementary file 2 — REPORTING SUMMARY [file 41541_2023_638_MOESM2_ESM.pdf]

nature portfolio

Corresponding author(s): Mangala Rao

Last updated by author(s): 2022/12/12

Reporting Summary

Nature Portfolio wishes to improve the reproducibility of the work that we publish. This form provides structure for consistency and transparency in reporting. For further information on Nature Portfolio policies, see our Editorial Policies and the Editorial Policy Checklist.

Statistics

For all statistical analyses, confirm that the following items are present in the figure legend, table legend, main text, or Methods section.

n/a	Confirmed	
☐	☒	The exact sample size (*n*) for each experimental group/condition, given as a discrete number and unit of measurement
☐	☒	A statement on whether measurements were taken from distinct samples or whether the same sample was measured repeatedly
☐	☒	The statistical test(s) used AND whether they are one- or two-sided<br>*Only common tests should be described solely by name; describe more complex techniques in the Methods section.*
☒	☐	A description of all covariates tested
☒	☐	A description of any assumptions or corrections, such as tests of normality and adjustment for multiple comparisons
☐	☒	A full description of the statistical parameters including central tendency (e.g. means) or other basic estimates (e.g. regression coefficient) AND variation (e.g. standard deviation) or associated estimates of uncertainty (e.g. confidence intervals)
☐	☒	For null hypothesis testing, the test statistic (e.g. *F*, *t*, *r*) with confidence intervals, effect sizes, degrees of freedom and *P* value noted<br>*Give P values as exact values whenever suitable.*
☒	☐	For Bayesian analysis, information on the choice of priors and Markov chain Monte Carlo settings
☒	☐	For hierarchical and complex designs, identification of the appropriate level for tests and full reporting of outcomes
☐	☒	Estimates of effect sizes (e.g. Cohen's *d*, Pearson's *r*), indicating how they were calculated

Our web collection on statistics for biologists contains articles on many of the points above.

Software and code

Policy information about availability of computer code

Data collection	Flow cytometry data was collected 5-laser Cytek Aurora flow cytometer (Cytek Biosciences) and BD LSRII (BD Biosciences). FluroSpot plates were counted and data nalyzed using the AID Autoimmune Diagnostica GmbH ELISpot reader.
Data analysis	Flow cytometry data was acquired by SpectroFlo software and BD FACS DIVA software and analyzed using Flow Jo software v 10 (BD), Graph Pad Prsim v. 9.4.1 was used for statistical analysis

For manuscripts utilizing custom algorithms or software that are central to the research but not yet described in published literature, software must be made available to editors and reviewers. We strongly encourage code deposition in a community repository (e.g. GitHub). See the Nature Portfolio guidelines for submitting code & software for further information.

Data

Policy information about availability of data

All manuscripts must include a data availability statement. This statement should provide the following information, where applicable:

- Accession codes, unique identifiers, or web links for publicly available datasets
- A description of any restrictions on data availability
- For clinical datasets or third party data, please ensure that the statement adheres to our policy

Data generated or analyzed during this study are included in this article and its supplementary information files.

Human research participants

Policy information about studies involving human research participants and Sex and Gender in Research.

Reporting on sex and gender	N/A
Population characteristics	N/A
Recruitment	N/A
Ethics oversight	N/A

Note that full information on the approval of the study protocol must also be provided in the manuscript.

Field-specific reporting

Please select the one below that is the best fit for your research. If you are not sure, read the appropriate sections before making your selection.

☒ Life sciences ☐ Behavioural & social sciences ☐ Ecological, evolutionary & environmental sciences

For a reference copy of the document with all sections, see nature.com/documents/nr-reporting-summary-flat.pdf

Life sciences study design

All studies must disclose on these points even when the disclosure is negative.

Sample size	In order to assure statistical significance in the results at-least N=5 mice/group/time point were taken and the experiments were repeated atleast twice independently. These have been described clearly in the methods section.
Data exclusions	No data were excluded
Replication	Experiments were repeated atleast twice independently and clearly explained in the methods. Each sample was run in triplicates for ELISA and FluoroSpot.
Randomization	All animals were randomly assigned to vaccination groups.
Blinding	Blinding was not performed, however the experiments were conducted in an unbiased manner to prevent potential biases in the experimental groups

Reporting for specific materials, systems and methods

We require information from authors about some types of materials, experimental systems and methods used in many studies. Here, indicate whether each material, system or method listed is relevant to your study. If you are not sure if a list item applies to your research, read the appropriate section before selecting a response.

Materials & experimental systems

n/a	Involved in the study	
☐	☒	Antibodies
☐	☒	Eukaryotic cell lines
☒	☐	Palaeontology and archaeology
☐	☒	Animals and other organisms
☒	☐	Clinical data
☒	☐	Dual use research of concern

Methods

n/a	Involved in the study	
☒	☐	ChIP-seq
☐	☒	Flow cytometry
☒	☐	MRI-based neuroimaging

Antibodies

Antibodies used	All the antibodies used in this study are described in the supplementary tables.
Validation	All the antibodies are validated by their respective manufacturer and were titrated prior to use

Eukaryotic cell lines

Policy information about cell lines and Sex and Gender in Research

Cell line source(s)	P388D1 cells (ATCC); mammalian Expi293 cells (Thermo Fisher Scientific); HEK293 (Integral molecular)
Authentication	Commercially purchased and previously used in several experiments.
Mycoplasma contamination	Negative for mycoplasma
Commonly misidentified lines (See ICLAC register)	None were utilized

Animals and other research organisms

Policy information about studies involving animals; ARRIVE guidelines recommended for reporting animal research, and Sex and Gender in Research

Laboratory animals	Female C57BL/6 mice (5–6 weeks of age) were obtained from The Jackson Laboratory
Wild animals	N/A
Reporting on sex	Female
Field-collected samples	N/A
Ethics oversight	Animal studies were carried out in accordance with the recommendations in the Guide for the Care and Use of Laboratory Animals of the National Institutes of Health.<br>The protocols were approved by the Institutional Animal Care and Use Committee at the Walter Reed Army Institute of Research [Assurance number D16-00596 (A4117-01)].

Note that full information on the approval of the study protocol must also be provided in the manuscript.

Flow Cytometry

Plots

Confirm that:

☐ The axis labels state the marker and fluorochrome used (e.g. CD4-FITC).

☒ The axis scales are clearly visible. Include numbers along axes only for bottom left plot of group (a 'group' is an analysis of identical markers).

☒ All plots are contour plots with outliers or pseudocolor plots.

☒ A numerical value for number of cells or percentage (with statistics) is provided.

Methodology

Sample preparation	Cryopreserved or fresh splenocytes or lymph node cells were used for the flow cytometry
Instrument	Cytek Aurora (Cytek Biosciences) and BD LSR II (BD Biosciences)
Software	SpectroFlo,<br>BD FACS DIVA,<br>FLOW JO
Cell population abundance	No sorting was performed
Gating strategy	provided in supplementary figures

☒ Tick this box to confirm that a figure exemplifying the gating strategy is provided in the Supplementary Information.
